# Toward the Analysis of Volatile Organic Compounds from Tomato Plants (*Solanum lycopersicum* L.) Treated with *Trichoderma virens* or/and *Botrytis cinerea*

**DOI:** 10.3390/cells12091271

**Published:** 2023-04-27

**Authors:** Justyna Nawrocka, Kamil Szymczak, Monika Skwarek-Fadecka, Urszula Małolepsza

**Affiliations:** 1 Department of Plant Physiology and Biochemistry, Faculty of Biology and Environmental Protection, University of Lodz, Banacha 12/16, 90-237 Lodz, Poland; 2 Institute of Natural Products and Cosmetics, Faculty of Biotechnology and Food Sciences, Lodz University of Technology, Stefanowskiego 2/22, 90-537 Lodz, Poland

**Keywords:** tomato defense responses, necrotroph, biocontrol

## Abstract

Gray mold caused by *Botrytis cinerea* causes significant losses in tomato crops. *B. cinerea* infection may be halted by volatile organic compounds (VOCs), which may exhibit fungistatic activity or enhance the defense responses of plants against the pathogen. The enhanced VOC generation was observed in tomato (*Solanum lycopersicum* L.), with the soil-applied biocontrol agent *Trichoderma virens* (10^6^ spores/1 g soil), which decreased the gray mold disease index in plant leaves at 72 hpi with *B. cinerea* suspension (1 × 10^6^ spores/mL). The tomato leaves were found to emit 100 VOCs, annotated and putatively annotated, assigned to six classes by the headspace GCxGC TOF-MS method. In *Trichoderma*-treated plants with a decreased grey mold disease index, the increased emission or appearance of 2-hexenal, (2E,4E)-2,4-hexadienal, 2-hexyn-1-ol, 3,6,6-trimethyl-2-cyclohexen-1-one, 1-octen-3-ol, 1,5-octadien-3-ol, 2-octenal, octanal, 2-penten-1-ol, (Z)-6-nonenal, prenol, and acetophenone, and 2-hydroxyacetophenone, β-phellandrene, β-myrcene, 2-carene, δ-elemene, and isocaryophyllene, and β-ionone, 2-methyltetrahydrofuran, and 2-ethyl-, and 2-pentylfuran, ethyl, butyl, and hexyl acetate were most noticeable. This is the first report of the VOCs that were released by tomato plants treated with *Trichoderma*, which may be used in practice against *B. cinerea*, although this requires further analysis, including the complete identification of VOCs and determination of their potential as agents that are capable of the direct and indirect control of pathogens.

## 1. Introduction

Volatile organic compounds (VOCs) are emitted by all plants from their leaves, flowers, fruits, and roots [1]. Chemically and structurally, VOCs belong to a large group of terpenoids (homo-, mono-, di-, and sesquiterpenoids), which are fatty acid-derived aliphatic hydrocarbons, including C6-volatiles and derivatives, aromatic compounds, and certain alkanes, alkenes, alcohols, esters, aldehydes, and ketones [1]. They are synthesized via several biochemical pathways [2]; for example, aliphatic oxylipins and green leaf volatiles (GLVs) are products of lipoxygenase (LOX) activity [3]; isoprenoids are generated by the deoxyxylulose-5-phosphate (DXP) and mevalonate (MVA) pathways for monoterpenes and sesquiterpenes, respectively [4,5]; and aromatic compounds, phenylpropanoids, and phenolics are synthesized via the phenylalanine ammonia-lyase (PAL) pathway, among others [1,6].

VOCs demonstrate considerable genotypic variation and phenotypic plasticity with regard to their content and composition. In addition, the quantity and quality of released VOCs can change dramatically following exposure to different biotic and abiotic stimuli, especially during the induction of systemic responses [2]. In plants, VOCs serve multiple functions. They are known as plant-to-plant communication molecules, as well as attractants for pollinators, seed dispersers, and other beneficial animals and microorganisms. VOCs are also intensively released in response to mechanical damage, insect feeding activity, pathogen infections, and abiotic stresses, such as drought and extreme temperatures [7]. It is suggested that as they are very active, effective at low concentrations, and can travel long distances, VOCs have an underestimated potential as plant growth promoters, pathogen inhibitors, and resistance inducers against biotic or abiotic stresses [8,9]. VOCs are important airborne signaling molecules that can boost different responses in neighboring plants and, together with other molecules, transmit signals within the plant [1].

VOCs play an important role in the communication of plants with microorganisms, which emit chemically diverse VOCs in the gas phase of a microbial culture [10]. Microbial VOCs (MVOCs) belong to a broad range of chemical classes, such as alcohols, carbonyl compounds, hydrocarbons, aromatic compounds, and sulfur- and nitrogen-containing compounds, and they show high structural variability [10]. Fungal volatiles are dominated by alcohols, benzenoids, aldehydes, alkenes, acids, esters, and ketones [11]. Microbial volatiles are considered products of primary and secondary metabolism, formed mainly by the oxidation of glucose from various intermediates [12,13]. The main function of the MVOCs is based on the interplay between microorganisms, generally between bacteria and fungi [14]. These interactions are often based on antagonism by MVOCs with antifungal activity, i.e., caryophyllene, hydrogen cyanide, 1-undecene, dimethyl disulphide, dimethyl trisulphide, S-methyl thioacetate, benzonitrile, etc., or antibacterial activity, i.e., γ-butyrolactones, α-flavenone, dihydro-β-agarofuran, 1- undecene, methanthiol, dimethyl disulfide, etc., although there may also be effective beneficial communication by such compounds, having an important role in interactions between physically separated microorganisms [12]. MVOCs can be of great benefit to plants and their use in agriculture thanks to their ability to inhibit the growth and development of plant pathogens, induce the activation of plant defenses, and promote plant growth and development [11]. On the other hand, MVOCs that are released by pathogens can negatively affect plant functioning [10].

Microbial infections can significantly affect the emission of VOCs by plants. Therefore, pathogen-induced changes in volatiles have been used for the early detection of disease incidence and the development of a defensive response to pathogens in crops [15]. The mechanisms by which VOCs affect infections in plants remain largely unknown; however, they are thought to act both as direct antipathogenic agents and signaling molecules in the plant’s defensive response [15,16]. VOCs are known to exert direct antipathogenic effects by various routes, including inhibiting ion transporting channels and enhancing reactive oxygen species (ROS) accumulation as well as by promoting mitochondrial fragmentation, chromatin condensation, and the disruption of enzymes’ conditioning pathogenicity [15]. However, these compounds have not received as much attention regarding defense responses and resistance induction as other signaling molecules, such as plant hormones and reactive oxygen and nitrogen species (ROS and RNS), and the role of different VOCs emitted by plants in the biocontrol of diseases needs further study. There is a clear need for a quantitative and qualitative analysis of plant-generated VOCs that may play an essential role in protecting against pathogens [17,18], since they may be implemented in future smart agricultural practices for plant protection and productivity [7]. *Botrytis cinerea* (teleomorph: *Botryotinia fuckeliana*) is an airborne phytopathogenic fungus that can infect more than 200 crop hosts worldwide and is ranked second in the world’s top 10 fungal plant pathogens list based on its scientific and economic importance [19,20]. The pathogen is a necrotroph, causing gray mold disease of leaves, stems, fruits, and flowers, both pre- and post-harvest [21,22]. One of the hosts of *B. cinerea* is tomato (*Solanum lycopersicum* L.), the first-ranked processing vegetable with a global cultivation area. Currently, no natural tomato cultivars demonstrate adequate resistance to *B. cinerea* infection. As such, the control strategies of *B. cinerea* are mainly based on the use of agrochemicals with harmful effects on both environmental and human health, or the use of transgenic tomato varieties. However, due to the varied self-protection strategies of the pathogen, including its high reproductive potential, ability to survive as sclerotia in crop debris in unfavorable conditions, and rapid genomic mutation toward resistance to fungicides, neither approach is sufficiently effective [19]. Therefore, considerable efforts have been made to develop safe and effective, complementary, or alternative methods of plant protection against *B. cinerea* [23,24]. The microbiological biocontrol has great potential to protect plants from *B. cinerea.* Several bacterial and fungal strains belonging to *Bacillus*, *Pseudomonas*, *Aureobasidium*, and *Trichoderma* spp. have been successfully selected as efficient biocontrol agents (BCAs) against *B. cinerea* through direct mechanisms, such as parasitism, antibiosis, and competition, but also indirectly through the activation of systemic plant defenses and resistance [24,25,26]. Regarding the latter, the effective systemic plant response against *B. cinerea* is related to (i) reduced oxidative damage and hydrogen peroxide (H_2_O_2_) accumulation; (ii) antagonistic interaction between the phytohormones, i.e., salicylic acid (SA) and jasmonic acid (JA) in which ethylene (ET) acts as a fine-tuning modulator; (iii) the enhancement of expression of defense-related genes, including PR-1, PR-2, PR-4, PR-5, PAL, NPR1, PDF1.2, COI1, LOX, and PINII; (iv) the induction of lipoxygenase (LOX), lipid hydroperoxidase (LHP), chitinase (CHI), β-1,3 glucanase, superoxide dismutase (SOD), catalase (CAT), glutathione S-transferase (GST), phenylalanine ammonia lyase (PAL), peroxidase (POX), and thaumatin-like protein activity; (v) callose deposition; and (vi) indolic derivative, phenolic compound, lignan, oxylipin, and phytoalexin accumulation in plants [26].

*Trichoderma* spp. are soilborne filamentous fungi, of which some strains are capable of establishing beneficial relationships with plants and acting as BCAs. *Trichoderma* spp. may promote growth in plants, protect them against abiotic stresses, and increase their level of protection against biotrophic and/or necrotrophic pathogens [27]. The beneficial influence exerted on plants depends on the *Trichoderma* strain, plant species, pathogen, and soil–climate conditions. Some *Trichoderma* strains are antagonists, competitors, or mycoparasites of pathogens, while others act indirectly by priming or enhancing defense responses and resistance in the plant [28,29]. *Trichoderma* strains are known to activate induced systemic resistance (ISR) and rarely, systemic acquired resistance (SAR). Recent studies also indicate the activation of a hybrid type of resistance, i.e., ISR/SAR, which is also known as TISR (*Trichoderma*-induced systemic resistance) [30,31]. However, the mechanism by which *Trichoderma* spp. induces defense responses and the type of resistance induced in plants still arouse much controversy [32,33,34].

Within filamentous fungi, *Trichoderma* spp. stands out as the main biocontrol agent against *B. cinerea* [30]. Selected *Trichoderma* strains were shown to directly suppress *B. cinerea* by competing for nutrients and colonization space [35] and by limiting mycelial growth and conidial germination of the pathogen, as a result of the activity of antifungal metabolites, hydrolytic enzymes, and VOCs [36,37]. There is a very wide diversity of secondary metabolites, including pyrones, butenolides, azaphylones, anthraquinones, trichothecenes, terpenoids, steroids, and peptaibols, which are produced by different *Trichoderma* spp. capable of inhibiting the growth and development of *B. cinerea* [25]. Some of the compounds released by *Trichoderma* spp. were shown to prime or elicit plant defense responses, which enhanced their protection against *B. cinerea* [38]. Regarding VOC emission by *Trichoderma* spp., different compounds have been suggested to be involved in plant protection against *B. cinerea*, especially hydrocarbon terpenes, including the sesquiterpenes β-caryophyllene, (−)-β-elemene, germacrene D, τ-cadinene, δ-cadinene, α-amorphene, and τ-selinene, and the monoterpenes β-myrcene, trans-β-ocimene, and cis-β-ocimene [39].

The defense responses and resistance of plants induced by *Trichoderma* spp. against *B. cinerea* are not fully characterized. However, in some studies carried out on *Trichoderma*–plant–*B. cinerea* interactions with *A. thaliana* and tomato plants, the responses of plants to microorganisms were shown to be dependent on the JA/ET or SA as well as ROS signaling pathways [26,40,41]. Recently, attention has been paid to the role of VOCs that are produced and emitted by plants in response to attacks by *B. cinerea* to develop new biopesticides [7,42]. Special attention is paid to the complex signaling networks related to *Trichoderma*-induced defense responses, which provide enhanced protection against *B. cinerea*; these seem to be based on the crosstalk between signaling molecules of resistance, including VOCs generated by plants [43]. While plant VOCs are known to play important roles in protecting plants against pathogens, their role in *Trichoderma*-induced resistance against various pathogens, including *B. cinerea*, remains relatively unclear.

In the present study, the designed experimental setup contains tomato plants, *Trichoderma virens* TRS 106 as BCA, and the pathogen *B. cinerea*. In the previous studies, TRS 106 was chosen from 25 *Trichoderma* isolates as the most effective in terms of tomato growth and development promotion, as well as in the reductions in incidences of *Rhizoctonia solani* by the stimulation of systemic resistance similar to TISR in tomato plants [44]. Moreover, TRS 106 presented the ability to protect tomato plants against *B. cinerea* by the induction of defense responses in plants related to nitric oxide (NO), ROS, and selected VOCs belonging to salicylates and GLVs signaling [45]. Based on the preliminary studies, we hypothesized that TRS 106 might induce the emission of other VOCs in tomato plants, which may be involved in defensive reactions providing plant protection against *B. cinerea*. Therefore, the primary and novel objective of the present work was to determine which VOC classes and specific VOCs are more strongly emitted during the defense responses of tomato plants against the pathogen *B. cinerea*, induced by *T. virens*, as, to the best of our knowledge, this has not yet been studied. The present study focuses on the analysis of VOCs from tomato plants treated with *T. virens* TRS 106 and/or *B. cinerea*.

## 2. Materials and Methods

### 2.1. Fungal Material

*T. virens* TRS 106 was obtained from the bank collection of the Microbiology Laboratory Department of Vegetable Plant Protection of the Research Institute of Horticulture (Skierniewice, Poland). The morphological identification and molecular classification of TRS 106 have been described previously by Oskiera et al. [46]. Isolate TRS 106 was chosen from 25 *Trichoderma* isolates as the most effective in promoting tomato growth and development and protecting against the pathogen *Rhizoctonia solani*, which was related to priming and induction in plant systemic resistance [44]. Before treating the tomatoes, TRS 106 was grown on malt extract agar medium for 10 days at 25 °C and exposed every 24 h to daylight for 20 min to activate fungus sporulation. To obtain TRS 106 inoculum, the spores of the fungus from one Petri plate were washed off the surface with 10 mL of 0.85% NaCl solution, and the inoculum was diluted with tap water to obtain 1 × 10^7^ spores/mL. *B. cinerea* isolate 1631, an effective pathogen of tomato plants, was obtained from the Bank of Plant Pathogens (Poznań, Poland) and was maintained in stock culture on potato dextrose agar (PDA) in the dark at 24 °C for 14 days. The conidial suspension was prepared by washing PDA cultures with tap water supplemented with 0.3 mM H_2_KPO_4_ and 2.2 mM glucose and diluted to obtain 1 × 10^6^ spores/mL.

### 2.2. Plant Material Cultivation, Inoculation with B. cinerea, and the Disease Development Determination

Six-week-old tomato plants (*S. lycopersicum* L.) belonging to two plant varieties, i.e., Perkoz and Remiz, were used in the experiment. The plant material, cultivation conditions, and inoculation with *B. cinerea* are conscientiously described previously [47]. Plants were grown in controlled conditions (one plant per pot with sowing potting soil and perlite 1:0.25 (*v*:*v*)). Ten days after sowing, the growing substrate of half of the tomato seedlings was supplemented with prepared TRS 106 spore suspension to obtain 10^6^ spore density per 1 g of the soil. The plants were cultivated for six weeks in a chamber at a temperature of 25/20 °C with a 14/10 h day/night photoperiod at 80% relative humidity. The light was supplied by white fluorescent lamps (type 36 W, Philips TDL 36/84) at 150 µEm^−2^s^−1^ intensity. After six weeks, the third fully expanded leaves on half of the control plants and half of the plants grown in the soil with TRS 106 were inoculated with a spore suspension of *B. cinerea* (1 × 10^6^ spores/mL). In each plant, the total adaxial surface of the area of one leaf was inoculated with 30 µL drops of *B. cinerea* suspension, with one drop per 2 cm^2^ of the leaf. The leaves of plants non-inoculated with *B. cinerea* were treated with drops of the medium used for the pathogen inoculum preparation (tap water supplemented with 0.3 mM H_2_KPO_4_ and 2.2 mM glucose). After inoculation, the tomato plants were placed into the chamber at a temperature of 20 °C and 80% relative humidity. The conditions were favorable to the *B. cinerea* infection development.

In the present experiment, the time interval between inoculation and sampling was 72 h. At that time, the leaves were cut off, photographed, and immediately used for the determination of VOCs. The diseased area on the leaves of tomato plants was measured using photographs processed in Motic Images Plus 2.0 ML (Motic China Group, Asia), according to the manufacturer’s instructions.

The disease development was scored using the following scale: 0 = no gray mold symptoms; 1 = gray mold symptoms up to 25% of the leaf area; 2 = gray mold symptoms from 25 to 50% of the leaf area; 3 = gray mold symptoms from 50 to 75% of the leaf area; and 4 = gray mold symptoms more than 75% of the leaf area. The disease index (DI) was calculated based on the results of the disease development scoring according to the formula described by Taheri and Tarighi [48], where DI = [(1n^1^ + 2n^2^ + 3n^3^ + 4n^4^)/4N] × 100%, with n1 as the number of plants with score 1, n^2^ as the number of plants with score 2, etc., and N as the total number of plants used in the treatment. The experiment was prepared three times under the same conditions with six replicates (plants) per treatment (n = 18).

In both tomato varieties, four experimental treatments of plants were tested:

(i) Plants grown in the soil without *T. virens* TRS 106 spores (control, C);
(ii) Plants grown in the soil with *T. virens* TRS 106 spores (TRS 106);
(iii) Plants grown in the soil without *T. virens* TRS 106 spores, and inoculated with *B. cinerea* (Bc);
(iv) Plants grown in the soil with *T. virens* TRS 106 spores, and inoculated with *B. cinerea* (TRS 106 + Bc).

### 2.3. Assay of VOC Emission

Continuing the experiment published in 2022 [47], and having enough plant material from the cultivations, we undertook a further, more detailed analysis of the emission of VOCs released by the leaves of tomato plants belonging to Perkoz and Remiz varieties, including (i) control plants, (ii) TRS 106 plants, (iii) Bc plants, and (iv) TRS 106 + Bc plants. In parallel with the study of VOC emission by tomato leaves, we also checked the emission of VOCs by the microorganisms alone (Supplementary Figure S1, Table S2), especially *B. cinerea*, which had direct contact with the leaves. Since TRS 106 was applied to the soil and its spores or hyphae were not detected in or on tomato shoots or leaves, the VOCs released by this strain do not pose a risk of contamination of the VOC blend emitted by the tomato leaves. The VOCs were determined by solid-phase microextraction (SPME), according to Carlin et al. [49], as described previously [45]. Three grams of freshly harvested leaves from a given experimental treatment were incubated in 20 mL headspace vials at 40 °C for 30 min. Subsequently, extraction was performed using 50/30 divinylbenzene/carboxen/polydimethylsilox (DVB/CAR/PDMS) 1 cm-long fiber for 60 min, and after that, the samples were introduced into gas chromatograph injection port and desorbed at 240 °C. GC-MS analyses were carried out on Pegasus 4D (LECO) apparatus, equipped with Agilent 6890N gas chromatograph coupled with time-of-flight mass spectrometer (LECO). Samples were injected with Gerstel Multi Purpose Sampler (MPS 2). The GC was fitted with a BPX5 capillary column of 30 m × 0.25 mm × 0.25 µm (SGE) for the first dimension and the BPX50 capillary column of 2 m × 0.1 mm × 0.1 µm (SGE) for the second dimension. Tomato leaves were analyzed in two dimensions (2D). Helium was used as a carrier gas with a flow rate of 1.2 mL/min. The inlet temperature was held at 240 °C and operated in splitless mode. The oven temperature was initially held at 35 °C for 5 min, ramped to 210 °C by 5 °C/min, and held for 5 min. The second dimension oven temperature program was 5 °C higher, respectively. The total run time was 45 min. The transfer line temperature was maintained at 250 °C. TOF mass spectrometer operated in EI mode, and parameters included mass range of m/z 33–550 at 30 spectra/s, ionization energy of 70 eV, and ion source temperature of 200 °C.

*B. cinerea* and *T. virens* TRS 106 cultures were analyzed quite similarly to the method described above; however, due to their less complex VOC profiles, the analyses were carried out in one dimension (1D). Despite the fact that the compounds were only separated in one column, all peaks were well split. Three grams of two-week-old *B. cinerea* or 10-day-old TRS 106 cultures used for inoculation were collected with a ceramic spatula and immediately transferred to 20 mL headspace vials at 40 °C for 30 min and then extracted using 50/30 DVB/CAR/PDMS 1 cm-long fiber for 60 min. The vials were glass, certified (Kinesis, Australia) with UltraClean Closure: 18 mm Magnetic Universal Screw Caps with silicon/PTFE septums (Kinesis, Australia). GC-MS apparatus operated at the same parameters as for the analyses of tomato leaves. The capillary column was BPX5 (30 m × 0.25 mm × 0.25 µm, SGE). Chromatograms were processed with ChromaTOF optimized for Pegasus 4D software. Data were processed in ChromaTOF version: 4.71.0.0 optimized for Pegasus with True Signal Deconvolution^®^. The NIST Mass Spectral Search Program for the NIST/EPA/NIH Mass Spectral Library Version 2.0 g was used. Peak integration parameters for 2DGC were as follows: baseline offset below 0.5 (through the middle of the noise); 1st dimension expected peak width 64; 2nd dimension expected peak width 0.8; match required to combine 50; minimum required S/N for the subpeak to be retained 5; and processing of peaks at minimum of S/N 800 throughout full run. For 1DGC, peak integration parameters were similar but with the following two differences: expected peak width 5 and processing of peaks at minimum of S/N 500 throughout full run. VOCs were determined based on a comparison of their mass spectra with those listed in the NIST and Wiley libraries databases. Furthermore, the Linear Retention Indices (LRIs) were calculated using a series of n-alkanes (C_8_–C_20_) and compared with the available retention data reported in the literature for the non-polar column (webbook.nist.gov, accessed on 1 September 2022). A compound was considered as annotated when the similarity with NIST database MS spectrum was greater than 800 (80%) and the RI (retention index) was close to the literature. Identifications were also confirmed by comparison of the retention times of the chromatographic peaks with those of selected co-injected commercial standards analyzed under the same conditions as well as by comparison to published GC profiles of tomato headspace volatiles [50,51,52,53]. Compounds that were not annotated using an authentic chemical standard, which was the strongest limitation of the chosen method, were considered putatively annotated according to the classification presented by [54] (Supplementary Figures S2 and S3; Tables S1 and S2). For individual volatiles, the peak area was calculated from the total ion chromatogram (TIC). Total VOCs belonging to different classes, i.e., alcohols, aldehydes, ketones, esters, terpenoids, and other VOCs, classified by the structure of compounds according to [1,55,56,57] were calculated by summing total GC peak areas of selected compounds. The graphic visualization of the dataset (100 VOCs) was prepared based on the results normalized by the value of the maximum peak area and presented as its respective % equivalents.

### 2.4. Statistical Analyses

After checking the normal distribution of the data (Shapiro–Wilk test) and the homogeneity of variances (Levene test), the effect of TRS 106 and *B. cinerea* treatments on each parameter was checked. Regarding DI evaluation, values represent the means and SE from three independent, not significantly different experiments (ANOVA, *α* ≥ 0.05) with six replicates (plants) per treatment (n = 18). Regarding DI, the statistical analysis of variance (one-way ANOVA, α ≤ 0.05) was followed by the Duncan multiple range post hoc test. Regarding the analysis of VOCs, values represent the means from five plants per treatment (n = 5). For each VOC class and each separate VOC, the non-parametric Kruskal–Wallis test (*p* ≤ 0.05) was performed. In both analyses, respective significant differences were marked using different letters (a, b, and c). All statistical evaluations were conducted using Statistica 13.1 software (StatSoft Inc., Tulsa, OK, USA).

## 3. Results

### 3.1. T. virens TRS 106 Reduces Gray Mold Disease Index in Tomato Plants

**Figure 1:**
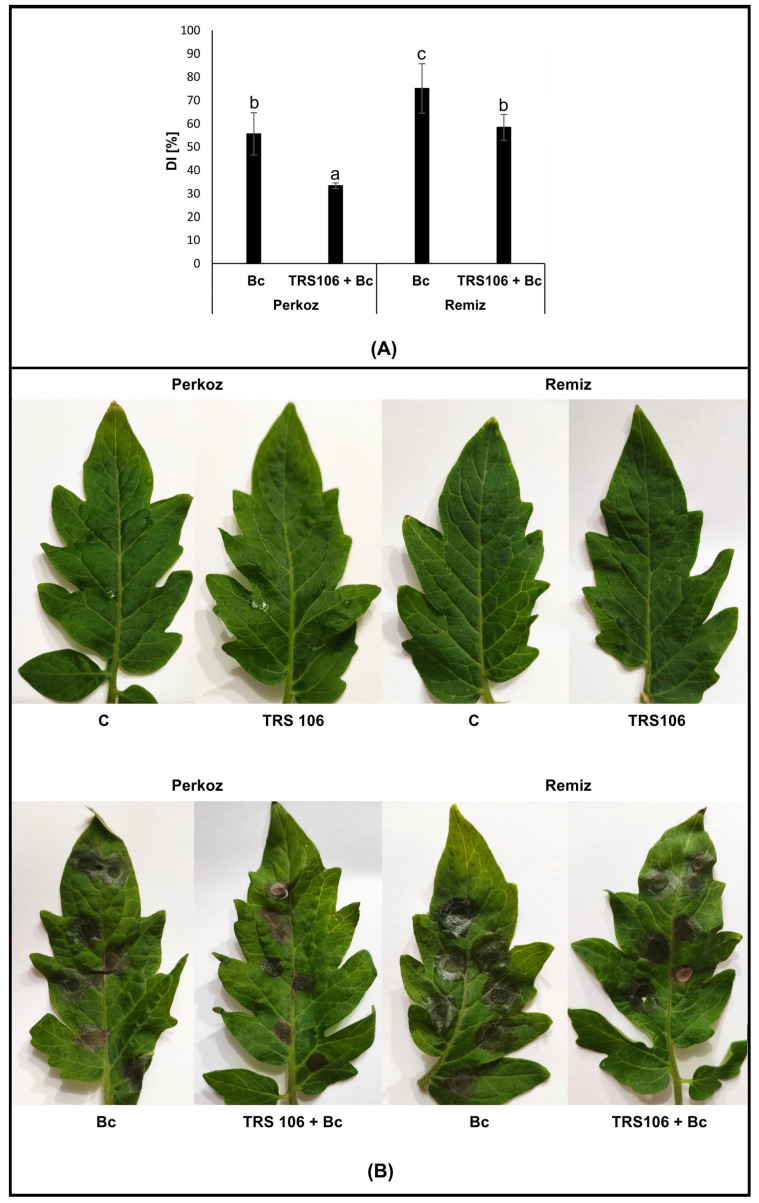
Evaluation of the gray mold disease index (DI) (**A**) together with disease symptoms identified as brown lesions, dark brown blight blotches, and rot on leaves (**B**), indicating disease severity 72 h after inoculation with *B. cinerea*. Values represent the means + SE from three independent, not significantly different experiments (ANOVA, α ≥ 0.05) with six replicates (plants) per treatment (n = 18). Regarding DI, the statistical analysis of variance (one-way ANOVA) was followed by the Duncan multiple range post hoc test. The data points followed by a different letter are significantly different at α ≤ 0.05. Abbreviations: C, control plants grown in the soil without TRS 106 spores; TRS 106, plants grown in the soil with TRS 106 spores; Bc, plants grown in the soil without TRS 106 spores, inoculated with *B. cinerea*; TRS 106 + Bc, plants grown in the soil with TRS 106 spores, inoculated with *B. cinerea*.

To confirm whether TRS 106 protects tomato plants against *B. cinerea*, the disease symptoms were compared between the leaves of Bc and Bc + TRS 106 plants. A successful infection by *B. cinerea* began within 48 h. The gray mold symptoms were identified by the presence of brown lesions, dark brown blight blotches, and rot symptoms on the leaves spreading irregularly from the pathogen inoculation sites (Figure 1). Seventy-two hours after inoculation, the infected area of the leaves was found to be greater in Remiz plants than Perkoz plants. Regarding disease development, we would like to emphasize that the results of the present studies are consistent with those obtained by us in previous studies [47], even if they were calculated using the results of measurements on leaves other than those tested previously. A reduced disease symptoms and pronounced decrease in the disease index (DI) were noted on the leaves of TRS 106 + Bc plants compared to the Bc plants, i.e., from 76% to 60% in Remiz and 55% to 36% in Perkoz plants, with the latter being the weakest observed symptoms of the disease.

### 3.2. Tomato Plants Belonging to Perkoz and Remiz Demonstrate Different Emission of VOCs

**Figure 2:**
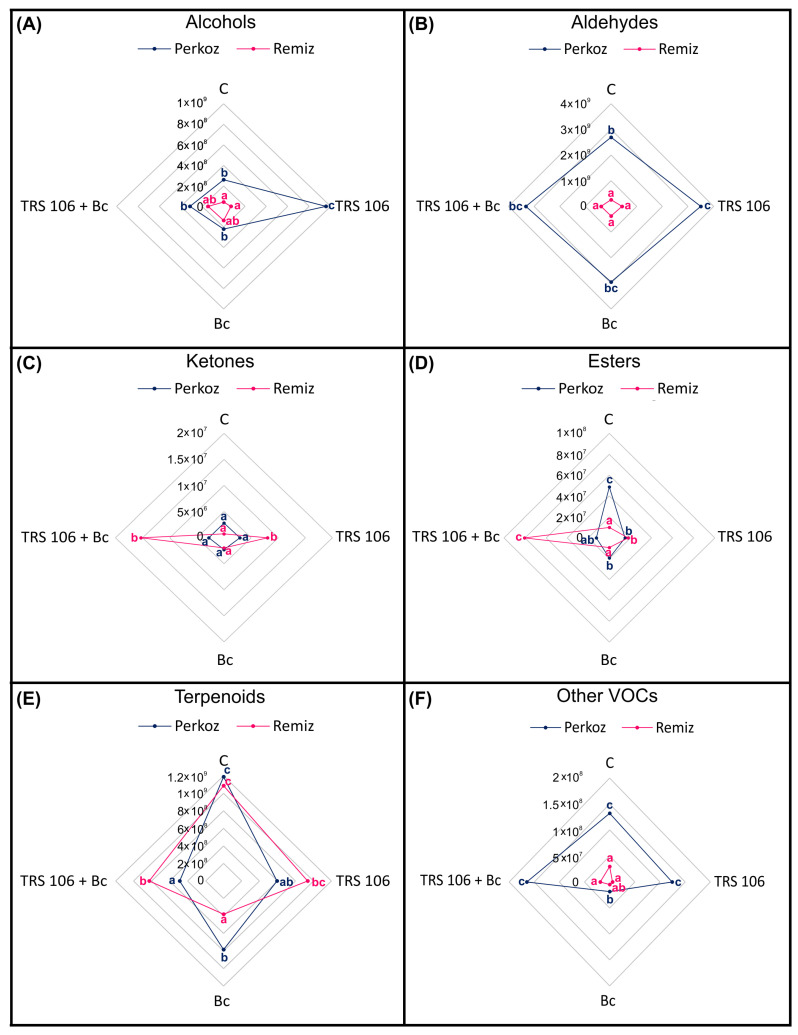
Differences in the total VOCs between experimental treatments; the VOCs belong to different classes, i.e., alcohols (**A**), aldehydes (**B**), ketones (**C**), esters (**D**), terpenoids (**E**), and other VOCs (**F**). Values represent the means from five plants per treatment (n = 5). For each VOC class, the non-parametric Kruskal–Wallis test (*p* ≤ 0.05) was performed. The data points followed by a different letter are significantly different at α ≤ 0.05. Abbreviations are as in Figure 1.

SPME coupled with GCxGC TOF-MS analysis presented 100 VOCs assigned to six classes, i.e., alcohols, aldehydes, ketones, esters, terpenoids, and other VOCs, which were all emitted by the leaves of tomato plants (Figure 2).

Depending on the possibility of an accurate detection, the compounds were labeled as annotated and putatively annotated (Table S1). The simultaneous analyses of *B. cinerea* and TRS 106 VOCs revealed several compounds that were not annotated in the VOCs of tomato leaves and several compounds where emissions were present in the VOCs of tomato leaves; however, the microorganisms released them at a much lower level (Supplementary Figure S1, Table S2).

**Figure 3:**
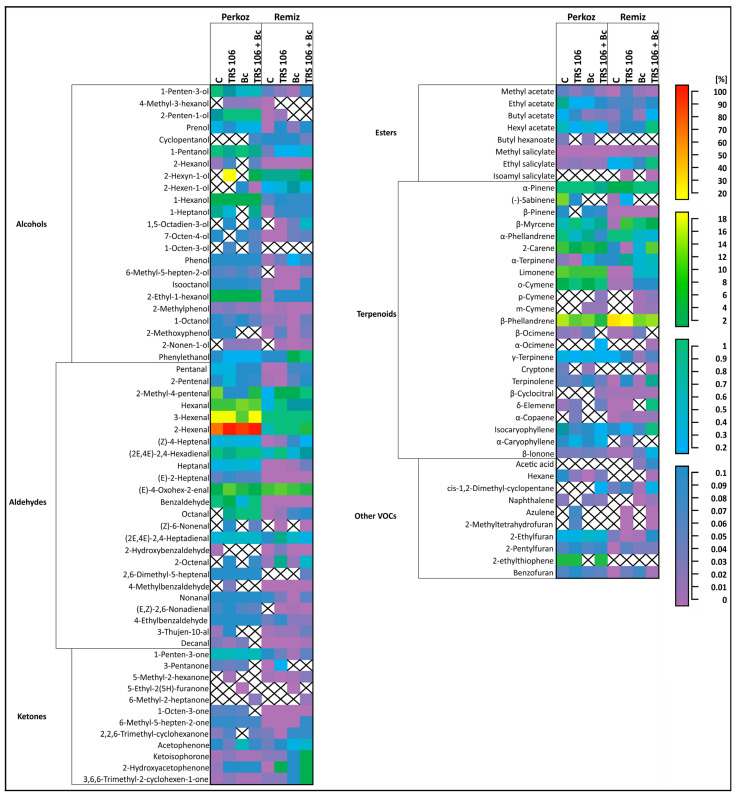
Selected compounds annotated and putatively annotated in the VOCs of tomato plants. The graphic visualization of the dataset (100 VOCs) was prepared based on the results normalized by the value of the maximum peak area and presented as its respective % equivalents.

The most heavily emitted VOC classes in both Perkoz and Remiz plants were alcohols, aldehydes, and terpenoids (Figure 2). Among aldehydes, the most strongly emitted compounds in Perkoz were 2- and 3-hexenal and 2-hexyn-1-ol. Among the terpenoids, β-phellandrene was the most strongly emitted compound in both Perkoz and Remiz (Figure 3, Table 1).

The crosses indicate the non-detection of the compound in the variant. The graphic visualization of the dataset (100 VOCs) was prepared based on the results normalized by the value of the maximum peak area. Abbreviations are featured in Figure 1.

After comparing all the tested treatments, Perkoz emitted more total aldehydes and other VOCs compared to Remiz (Figure 2). Additionally, compared to the control Remiz, the control Perkoz emitted more total alcohols, esters, and other VOCs. Comparing the Bc treatments, Perkoz emitted more esters, terpenoids, and other VOCs compared to Remiz. Compared to the TRS 106 Remiz, the TRS 106 Perkoz emitted more alcohols and other VOCs and fewer ketones, and compared to the TRS 106 + Bc Remiz, the TRS 106 + Bc Perkoz emitted fewer ketones, esters, and terpenoids (Figure 2).

### 3.3. VOCs in Tomato Plants, Belonging to the Perkoz and Remiz Varieties, and Infected with B. cinerea

To confirm whether the treatment of tomato leaves with *B. cinerea* influences the emission of VOCs, the compounds determined in the Bc plants were compared with those of the respective controls. In Bc Perkoz and Remiz, *B. cinerea* did not increase the total emission of all the VOC classes (Figure 2). However, in Bc Perkoz, as compared to the respective controls, pathogen treatment caused an increase in the emissions of single compounds, such as (2E,4E)-2,4-hexadienal, β-ocimene, and ethylfuran; it also stimulated the appearance of 2-methyl-3-hexanol, 2-hexen-1-ol, 2-nonen-1-ol, octanal, 5-ethyl-2(5H)-furanone, and m-cymene (Figure 3, Table 1).

In Bc Remiz, *B. cinerea* increased the levels of the emissions of various individual compounds, including 2-hexen-1-ol, phenol, and phenylethanol among alcohols; 2,2,6-trimethyl-cyclohexanone, and acetophenone among ketones; methyl salicylate among the esters; β-pinene, o-cymene, β-ocimene, α-copaene among terpenoids; and benzofuran as compared to the control; it also stimulated the appearance of other alcohols, i.e., 1,5-octadien-3-ol, 6-methyl-5-hepten-2-ol, and 2-nonen-1-ol, aldehyde (E,Z)-2,6-nonadienal, ketone 5-ethyl-2(5H)-furanone, terpenoids, i.e., p- and m-cymene, and other VOCs, i.e., acetic acid, and naphthalene (Figure 3, Table 1).

### 3.4. VOCs in Tomato Plants Treated with T. virens TRS 106 and Uninoculated with B. cinerea

To determine whether the application of *T. virens* to the soil influences the emission of VOCs from the tomato leaves, the VOCs observed in the TRS 106 plants were compared with those of their respective controls. In TRS 106 Perkoz plants, *T. virens* caused significant increases in the total alcohol and aldehyde contents and in TRS 106 Remiz ketone and ester contents compared to the respective controls (Figure 2).

Regarding the alcohols in TRS 106 Perkoz, *T. virens* caused enhanced emissions of 2-hexanol and 2-ethyl-1-hexanol and induced the appearance of 4-methyl-3-hexanol, 2-hexyn-1-ol, 1,5-octadien-3-ol, 1-octen-3-ol, and 2-nonen-1-ol (Figure 3, Table 1). Regarding the aldehydes in TRS 106 Perkoz, *T. virens* caused enhanced emissions of pentenal, 2-hexenal, (E)-2-heptenal, (E)-4-oxohex-2-enal, and 3-thujen-10-al, and induced the appearance of octanal, (Z)-6-nonenal, 2-octenal, and 4-methylbenzaldehyde (Figure 3, Table 1). In addition, TRS 106 Perkoz demonstrated increased emissions of methyl salicylate and the appearance of 5-methyl-2-hexanone, α-copaene, azulene, and 2-methyltetrahydrofuran (Figure 3, Table 1).

Regarding the ketones in TRS 106 Remiz, *T. virens* caused enhanced emissions of 3-pentanone, 1-octen-3-one, acetophenone, and 2-hydroxyacetophenone, and regarding esters, *T. virens* caused enhanced emissions of methyl and butyl acetate and methyl salicylate, and induced the appearance of isoamyl salicylate (Figure 3, Table 1). In addition, TRS 106 Remiz demonstrated increased emissions of various other compounds, such as prenol, 2-ethyl-1-hexanol, 2-methylphenol, 2-methoxyphenol, (2E,4E)-2,4-hexadienal, (2E,4E)-2,4-heptadienal, 2-octenal, decanal, β-pinene, β-myrcene, α-terpinene, β-cyclocitral, α-copaene, 2-pentylfuran, and benzofuran, as compared to the respective control. It also demonstrated the appearance of 1,5-octadien-3-ol, 6-methyl-5-hepten-2-ol, 2-nonen-1-ol, (Z)-6-nonenal, (E,Z)-2,6-nonadienal, α-caryophyllene, azulene, and 2-methyltetrahydrofuran (Figure 3, Table 1).

### 3.5. VOCs in Tomato Plants Treated with T. virens TRS 106 and Inoculated with B. cinerea

To confirm whether the application of *T. virens* to the soil influences the emission of VOCs from tomato leaves inoculated with *B. cinerea*, the VOCs produced in TRS 106 + Bc plants were compared with their respective controls. The TRS 106 + Bc Perkoz plants demonstrated enhanced emissions of several alcohols, i.e., 2-penten-1-ol and 2-hexanol, aldehyde (2E,4E)-2,4-hexadienal, and ester methyl salicylate compared to the respective controls; it also stimulated the appearance of other alcohols, i.e., 4-methyl-3-hexanol, cyclopentanol, 2-hexyn-1-ol, 2-hexen-1-ol, 1,5-octadien-3-ol, 1-octen-3-ol, 2-nonen-1-ol, aldehydes, i.e., octanal, (Z)-6-nonenal, 2-octenal, ketones, i.e., 6-methyl-2-heptanone, ketoisophorone, and terpenoids, i.e., p- and m-cymene, and α-ocimene (Figure 3, Table 1).

TRS 106 + Bc Remiz demonstrated enhanced emissions of total ketones and esters (Figure 2). In the case of ketones in TRS 106 + Bc Remiz plants, enhanced emissions of 5-methyl-2-hexanone, 2,2,6-trimethyl-cyclohexanone, acetophenone, 2-hydroxyacetophenone, and 3,6,6-trimethyl-2-cyclohexen-1-one compared to the respective controls were observed; the strain also stimulated the appearance of 6-methyl-2-heptanone (Figure 3, Table 1). Regarding esters in TRS 106 + Bc Remiz plants, enhanced emissions of ethyl, butyl, and hexyl acetate as well as methyl and ethyl salicylate were observed; it also stimulated the appearance of isoamyl salicylate. Moreover, TRS 106 + Bc Remiz demonstrated increased emissions of miscellaneous single compounds, such as prenol, 1-pentanol, 7-octen-4-ol, phenol, isooctanol, and phenylethanol among alcohols, 2-hexenal, (Z)-4-heptenal, heptanal, 2-octenal, and decanal among aldehydes, β-pinene, β-myrcene, 2-carene, α-terpinene, δ-elemene, α-copaene, isocaryophyllene, and β-ionone among terpenoids and cis-1,2-dimethyl-cyclopentane among other VOCs. Additionally, in TRS 106 + Bc Remiz the appearance of 1,5-octadien-3-ol, 6-methyl-5-hepten-2-ol, 2-nonen-1-ol, (Z)-6-nonenal, 2,6-dimethyl-5-heptenal, (E,Z)-2,6-nonadienal, p- and m-cymene, α-ocimene, cryptone, acetic acid, hexane, naphthalene, azulene, and 2-methyltetrahydrofuran non-detectable in the control plants, was observed (Figure 3, Table 1).

## 4. Discussion

The ability of *Trichoderma* spp. to induce plant defense responses and systemic resistance is essential for plant protection against a wide spectrum of viral, bacterial, and fungal pathogens [40,58]. Common plant diseases, such as rot, damping off, and wilt, were shown to be controlled by *Trichoderma* spp., which acts by killing or suppressing pathogens, promoting plant growth and development, inducing mechanical barriers in plants, and enhancing of plant defense responses and systemic resistance to pathogens [58]. Several *Trichoderma* strains have been found to protect plants against *B. cinerea* [24]. For example, antagonistic *T. harzianum* reduced *B. cinerea* germination and growth [59], while *T. harzianum*, *T. koningiopsis*, and *T. hamatum* induced the systemic resistance of tomato plants against the pathogen [23,60].The results of Mathys et al. [60] showed that in *A. thaliana*, *T. hamatum*-induced resistance against *B. cinerea*, at the molecular level, was related to the signaling of SA and the non-expressor of pathogenesis-related gene 1 (NPR1). Additionally, at a later stage of defense, it was based on JA signaling and the enhanced production of ROS, anthocyanins, flavonoids, and galactolipids. Regarding the role of VOCs in plant protection by *Trichoderma* against different pathogens, most of the work has focused on MVOCs released by the microorganism, which have been shown to promote plant growth via improved photosynthesis rates, increased plant resistance to pathogens, and activated phytohormone signaling pathways [61]. Regarding plant VOCs, their role in defense responses and resistance induced by *Trichoderma* against different pathogens, including *B. cinerea*, has only been elucidated to a small extent. However, there are a growing number of characteristics of VOC profiles that are changed in plants treated with *Trichoderma*. For example, Battaglia et al. [62] demonstrated that tomato plants whose roots were colonized by *T. longibrachiatum* showed quantitative differences in the release of specific VOCs, and as presented by Dini et al. [63], *T. harzianum* and *T. asperellum* differentially enhanced VOC production, affecting three biosynthetic pathways: methylerythritol 1-phosphate (MEP), lipid-signaling, and shikimate pathways in olive trees (*Olea europaea* L.).

The present studies confirmed that *T. virens* TRS 106 significantly reduced the DI of gray mold caused by *B. cinerea* in tomato plants belonging to Perkoz and Remiz varieties. These results are consistent with the results obtained by us in previous studies, which revealed the enhanced emission of aromatic and GLV VOCs, including salicylate and hexanol derivatives, by plants treated with *T. virens* TRS 106 and significantly protected against *B. cinerea* [47]. In the present studies, the detection of 100 VOCs belonging to different classes, i.e., alcohols, aldehydes, ketones, esters, terpenoids, and others, as well as the selection of several of them that may be taken into account in further research on the biocontrol of *B. cinerea,* is a novelty concerning previous research in this area, as well as the results presented in [47]. Hereby, we annotated 76 compounds that were not presented in our previous studies. We do not exclude the fact that some of the compounds may be derivatives or secondary metabolites that could be released accidentally; however, our findings also identify the VOCs that had a positive influence on the plants, reflected in a reduction in DI as a result of TRS 106 treatment.

Our findings showed that compared to Remiz, Perkoz plants are characterized by stronger emissions of VOCs, including aldehydes in all treatments, alcohols in the control and TRS 106 Perkoz plants, esters in the control and Bc Perkoz plants, and terpenoids in Bc Perkoz plants. On the other hand, Remiz plants treated with TRS 106 released more ketones, esters, and terpenoids than TRS 106 + Bc plants. Regarding the comparison of innate defense in Bc plants and induced defense in TRS 106 + Bc plants, which may point to potential VOCs usable to counter *B. cinerea* infection, TRS 106 + Bc Perkoz demonstrated stronger emissions of compounds included in other VOCs, including different alkanes, aromatics, and heterocyclic compounds compared to Bc, and TRS 106 + Bc Remiz demonstrated stronger emissions of total ketones and terpenoids. Based on the obtained results, it is expected that *T. virens* had a stronger influence on VOC emissions in Remiz plants because these plants naturally release fewer VOCs than Perkoz. However, such a statement requires much further research. The differences in VOC emissions between Perkoz and Remiz are not surprising since it is well known that plant functionality in general strongly depends on the genotype, and even small genomic differences may influence the response of a variety to different stimuli [24,64].

It has been demonstrated that VOCs influence attacks by pathogens in various ways. For example, some VOCs emitted by cherry tomato and strawberry fruits facilitate *B. cinerea* infection [65,66]; however, more reports suggest that VOCs play protective roles against this pathogen [67,68]. These compounds are involved both in direct and indirect defense systems, where they inhibit the spread of the pathogen into plant tissues while also playing an important role as defense response signaling molecules [1]. Therefore, our further analysis examined which compounds belonging to the mentioned classes were emitted by plants that were less infected with *B. cinerea*.

In the present studies, the biggest VOC classes are alcohols and aldehydes, which, together with ketones, contain different aliphatic and cyclic hydrocarbons, fatty acid derivatives, and GLVs. Aliphatic C_5_–C_10_ hydrocarbons are fatty acid-derived VOCs that play many important roles in plant functioning, including defense responses [69,70,71]. Regarding the C_6_ volatiles, which are considered important elicitors and markers of plant defense responses to stress [72], in the present studies, special attention should be paid to (2E,4E)-2,4-hexadienal and 2-hexyn-1-ol as well as 2-hexenal and 3,6,6-trimethyl-2-cyclohexen-1-one, which were strongly emitted, respectively, by Perkoz or Remiz plants with lowered gray mold DI. In addition, the TRS 106 + Bc plants, which were characterized by lowered gray mold DI than the Bc plants, intensively emitted more alcohols, aldehydes, and ketones, including 1,5-octadien-3-ol, 2-octenal, and (Z)-6-nonenal in both Perkoz and Remiz; 2-penten-1-ol, 1-octen-3-ol, and octanal in Perkoz; as well as prenol, acetophenone, and 2-hydroxyacetophenone in Remiz. Some of these compounds were absent in the Bc plants and plants not treated with *T. virens.*

Some of the alcohols, aldehydes, or ketones annotated and putatively annotated in the TRS 106-treated plants, or similar ones, have been previously presented as active molecules against different pathogens. For example, 2-hexenal was reported to be effective in the suppression of growth and germination of the pathogen *Monilinia laxa*, and was found to protect apricot, nectarine, and peach against brown rot of the fruit as a post-harvest biofumigant [73]. In addition, 1-octen-3-ol, 2,6-dimethyl-2,4,6- octatriene, nonanal, and trans-2-decenal were found to inhibit the growth of various pathogenic fungi, including *Colletotrichum lindemuthianum*, *B. cinerea*, and *Fusarium oxysporum* [16]. In addition, 2-hexanal and 2-nonenal demonstrated strong fungistatic properties against *B. cinerea* [74]. Regarding *Trichoderma*, Dini et al. [63] showed that in olive trees (*Olea europaea* L.), *T. harzianum* and *T. asperellum* enhanced nonanal formation by regulating the lipid-signaling pathway.

The other prevalent compounds annotated in the present study are the terpenoids. These compounds represent one of the most abundant and varied classes of VOCs derived from terpenes [75] and have been shown to play an important role in plant adaptive responses to biotic and abiotic stresses [76]. Our present findings indicate that β-phellandrene predominated among all the annotated terpenoids emitted by both Perkoz and Remiz; however, its emission was not stimulated by TRS 106. Nevertheless, many of the other annotated and putatively annotated terpenoids seemed to appear or be emitted more intensively by TRS 106 + Bc plants, which showed lower gray mold DI than Bc. The positive influence of TRS 106 on terpenoid emission was observed especially in Remiz plants exhibiting the enhanced emission of β-myrcene, 2-carene, δ-elemene, isocaryophyllene, and β-ionone. The ability of *Trichoderma* spp. to enhance terpenoid biosynthesis by controlling the MEP pathway was suggested previously [63]. Various volatile or semivolatile terpenoids, including low-molecular-weight monoterpenoids, diterpenoids, and sesquiterpenoids, have been found to have anti-phytopathogen properties, both above and below ground [77]. For example, (E)-β-caryophyllene emitted by *A. thaliana* was shown to be involved in plant protection against *P. syringae* [78], and (+)-3-carene in *Picea sitchensis* was associated with resistance to white pine weevil (*Pissodes strobi*) [79]. Some isoprenoids have also demonstrated signaling functions; for example, dehydroabietinal, which is produced in *Arabidopsis* leaf tissue, serves as a vascular signaling compound and a potent activator of SAR [80].

*B. cinerea* was reported to be sensitive to the in vitro application of (+)-limonene, (+)-carvone, citral, L-linalool, nerolidol, eugenol, and p-cymene [16,81,82]. In addition, the sesquiterpenes β-caryophyllene, (−)-β-elemene, germacrene D, τ-cadinene, δ-cadinene, α-amorphene, and τ-selinene and the monoterpenes β-myrcene, trans-β-ocimene, and cis-β-ocimene released by *T. virens* enhanced *A. thaliana* development and elicited defense responses against *B. cinerea* [39]. However, while various isoprenoids have been found to have direct fungistatic properties, the mechanisms they use to enhance the plant’s defensive response against *B. cinerea* remain poorly understood.

Aromatic compounds are abundant and structurally diverse in plants [83]. This group includes benzaldehyde and phenol derivatives and salicylates, which are important molecules that act as endogenous signals to trigger plant defense responses related to, inter alia, ROS production and pathogenesis-related (PR) gene expression [84]. A detailed analysis of aromatic compound emissions and their potential functions during the defense reactions of tomato plants to *B. cinerea* is given elsewhere [47].

An interesting group of annotated and putatively annotated compounds are volatile furan derivatives belonging to the other VOC class. These compounds are known to act as growth-promoting agents and are recommended for commercial use in agriculture to improve and control plant health [85]. The ability of furans to induce defense responses against pathogens is still poorly studied, but several reports have highlighted their remarkable inactivating, protective, and curative activities against plant viruses, e.g., tobacco mosaic virus [86], and bacteria, such as *Xanthomonas oryzae*, which is responsible for bacterial leaf blight [87]. In the present study, the emission of furan derivatives, including 2-methyltetrahydrofuran, 2-ethyl-, and 2-pentylfuran, was positively influenced by *T. virens* in TRS 106 + Bc Remiz, suggesting that they may be another group of components participating in protecting tomato plants against *B. cinerea*. Regarding other VOCs, another important compound that is strongly released by Perkoz plants, except for the Bc treatment, is 2-ethyltiophene, a thiophene derivative with a known negative impact on plant pathogens [88].

The last group of VOCs annotated and putatively annotated in tomato plants are the volatile derivatives of acetic acid, belonging to the class of esters. These compounds were generally more strongly released by Perkoz than Remiz, except for the TRS 106 + Bc treatment, where TRS 106 enhanced emissions of ethyl, butyl, and hexyl acetate. Acetic acid is one of the most multifunctional compounds in plants. It has been shown to successfully protect plants under stress conditions, for example, by enhancing leaf turgor, supporting photosynthesis, reducing oxidative stress, and enhancing the antioxidative system [89]. Regarding the acetate esters, butyl and hexyl acetates were shown to play a dual role in plant interaction with pathogens. For example, hexyl acetate appeared to stimulate conidial adhesion of *B. cinerea* strains to the *Vitis vinifera* grape fruit skin, which might facilitate fungal colonization [90]. In contrast, butyl acetate emitted by *Trichoderma* spp. affected the morphology and mycelial development of *Colletotrichum gloeosporioides*, thereby inhibiting radial growth, reducing spore formation, and inducing soft colonies [91].

In summary, the present studies confirmed that *T. virens* TRS 106, a defined biocontrol agent of *B. cinerea*, effectively decreased the DI of gray mold in tomato plants belonging to Perkoz and Remiz and showed a stimulating influence on the total volatile profiles as well as individual VOCs of both varieties. Special attention should be paid, for example, to 2-hexenal, (2E,4E)-2,4-hexadienal, 2-hexyn-1-ol, 3,6,6-trimethyl-2-cyclohexen-1-one, 1-octen-3-ol, 1,5-octadien-3-ol, 2-octenal, octanal, 2-penten-1-ol, (Z)-6-nonenal, prenol, and acetophenone; and 2-hydroxyacetophenone, β-phellandrene, β-myrcene, 2-carene, δ-elemene, and isocaryophyllene; and β-ionone, 2-methyltetrahydrofuran, and 2-ethyl- and 2-pentylfuran, ethyl, butyl, and hexyl acetate, whose emission increased or appeared in *Trichoderma* treated Perkoz, Remiz, or both plants with decreased DI of gray mold.

The presented results encourage further, thorough identification of selected VOCs emitted by tomato plants, which is emphasized in the summary, as well as the determination of the following: (i) what are the exact, real concentrations of selected VOCs emitted by tomato plants in different environmental conditions, especially those favorable to *B. cinerea* infection development; (ii) what is the direct influence of annotated VOCs and different blends of VOCs on *B. cinerea*; and (iii) whether the compounds show fungistatic potential against other pathogens of tomato plants.

From a practical point of view, our obtained results shed further light on the protective influence of TRS 106 on tomato plants, resulting in greater protection against *B. cinerea*. In our opinion, the potential inhibitory effect of the newly annotated and putatively annotated VOCs released by plants against *B. cinerea* deserves further in-depth analysis. These VOCs may enlarge the pool of compounds that have the potential to be used in integrated agriculture aimed at protecting plants against gray mold disease.

## Figures and Tables

**Table 1:** Selected compounds annotated and putatively annotated in the VOCs of tomato plants. Values represent the means from five plants per treatment (n = 5). For each separate VOC, the non-parametric Kruskal–Wallis test (*p* ≤ 0.05) was performed. The data points followed by a different letter are significantly different at *p* ≤ 0.05. Abbreviations are featured in Figure 1.

VOC	Perkoz				Remiz			
	C	TRS 106	Bc	TRS 106 + Bc	C	TRS 106	Bc	TRS 106 + Bc
Alcohols								
1-Penten-3-ol	4.3 × 10^7^ ± 4.1 × 10^6 (c)^	1.8 × 10^7^ ± 1.5 × 10^6 (b)^	1.3 × 10^7^ ± 1.8 × 10^6 (b)^	1.6 × 10^7^ ± 3.5 × 10^6 (b)^	1.5 × 10^6^ ± 7.6 × 10^4 (a)^	8.9 × 10^5^ ± 2.9 × 10^4 (a)^	3.4 × 10^5^ ± 1.9 × 10^4 (a)^	2.4 × 10^6^ ± 3.5 × 10^5 (a)^
4-Methyl-3-hexanol	nd	8.5 × 10^5^ ± 3.7 × 10^4 (a)^	8.5 × 10^5^ ± 3.3 × 10^4 (a)^	6.5 × 10^5^ ± 1.9 × 10^4 (a)^	nd	nd	nd	nd
2-Penten-1-ol	1.8 × 10^7^± 2.4 × 10^6 (b)^	1.5 × 10^7^ ± 7.0 × 10^6 (b)^	2.8 × 10^7^ ± 8.5 × 10^6 (bc)^	3.4 × 10^7^ ± 4.5 × 10^6 (c)^	1.3 × 10^5^ ± 4.1 × 10^4 (a)^	6.3 × 10^5^ ± 1.2 × 10^5 (a)^	nd	nd
Prenol	7.7 × 10^6^ ± 6.8 × 10^5 (bc)^	4.4 × 10^6^ ± 5.8 × 10^5 (b)^	7.8 × 10^6^ ± 1.0 × 10^6 (bc)^	9.0 × 10^6^ ± 4.2 × 10^5 (c)^	1.4 × 10^5^ ± 3.2 × 10^4 (a)^	4.6 × 10^6^ ± 1.7 × 10^5 (b)^	8.0 × 10^5^ ± 4.7 × 10^4 (a)^	4.6 × 10^6^ ± 2.5 × 10^5 (b)^
Cyclopentanol	nd	nd	nd	2.1 × 10^6^ ± 1.8 × 10^5 (ab)^	3.3 × 10^6^ ± 4.6 × 10^5 (ab)^	3.4 × 10^6^ ± 5.3 × 10^5 (ab)^	4.6 × 10^6^ ± 3.4 × 10^5 (b)^	1.4 × 10^6^ ± 4.0 × 10^5 (a)^
1-Pentanol	3.5 × 10^7^ ± 3.9 × 10^6 (c)^	2.4 × 10^7^ ± 1.8 × 10^6 (bc)^	2.8 × 10^7^ ± 5.0 × 10^6 (bc)^	2.1 × 10^7^ ± 4.9 × 10^6 (bc)^	1.4 × 10^6^ ± 8.4 × 10^5 (a)^	6.2 × 10^6^ ± 2.1 × 10^5 (ab)^	5.7 × 10^6^ ± 4.4 × 10^5 (ab)^	1.1 × 10^7^ ± 8.4 × 10^4 (b)^
2-Hexanol	6.3 × 10^5^ ± 2.3 × 10^4 (b)^	2.1 × 10^6^ ± 8.6 × 10^4 (c)^	nd	1.6 × 10^6^ ± 3.9 × 10^5 (c)^	1.1 × 10^5^ ± 8.0 × 10^3 (a)^	2.3 × 10^5^ ± 1.8 × 10^4 (a)^	1.3 × 10^5^ ± 2.9 × 10^4 (a)^	1.7 × 10^5^ ± 1.6 × 10^4 (a)^
2-Hexyn-1-ol	nd	7.2 × 10^8^ ± 1.7 × 10^7 (b)^	nd	6.6 × 10^7^ ± 3.8 × 10^6 (a)^	2.3 × 10^7^ ± 1.2 × 10^6 (a)^	2.4 × 10^7^ ± 3.0 × 10^6 (a)^	2.3 × 10^7^ ± 2.4 × 10^6 (a)^	5.2 × 10^7^ ± 8.4 × 10^5 (a)^
2-Hexen-1-ol	nd	nd	2.7 × 10^6^ ± 2.6 × 10^5 (b)^	1.5 × 10^5^ ± 1.0 × 10^4 (a)^	6.0 × 10^6^ ± 4.7 × 10^5 (b)^	1.1 × 10^7^ ± 9.4 × 10^4 (abc)^	1.8 × 10^7^ ± 3.9 × 10^6 (c)^	7.2 × 10^6^ ± 1.3 × 10^5 (b)^
1-Hexanol	5.9 × 10^7^ ± 3.5 × 10^6 (b)^	5.4 × 10^7^ ± 5.4 × 10^6 (b)^	5.5 × 10^7^ ± 5.0 × 10^6 (b)^	6.1 × 10^7^ ± 9.5 × 10^5 (b)^	1.6 × 10^6^ ± 4.6 × 10^4 (a)^	2.6 × 10^6^ ± 3.1 × 10^5 (a)^	2.3 × 10^6^ ± 2.4 × 10^5 (a)^	2.4 × 10^6^ ± 3.0 × 10^5 (a)^
1-Heptanol	2.4 × 10^7^ ± 2.7 × 10^6 (c)^	1.1 × 10^7^ ± 1.3 × 10^6 (b)^	nd	2.3 × 10^7^ ± 4.1 × 10^6 (c)^	2.3 × 10^7^ ± 3.6 × 10^6 (a)^	2.2 × 10^6^ ± 3.1 × 10^5 (ab)^	2.3 × 10^6^ ± 1.1 × 10^5 (ab)^	2.4 × 10^6^ ± 4.9 × 10^5 (ab)^
1,5-Octadien-3-ol	nd	2.8 × 10^6^ ± 5.0 × 10^5 (b)^	nd	3.4 × 10^6^ ± 5.4 × 10^5 (b)^	nd	1.5 × 10^5^ ± 3.8 × 10^4 (a)^	1.2 × 10^6^ ± 2.6 × 10^5 (b)^	1.2 × 10^7^ ± 2.1 × 10^6 (c)^
7-Octen-4-ol	3.6 × 10^6^ ± 6.7 × 10^5 (b)^	nd	2.2 × 10^6^ ± 3.8 × 10^5 (b)^	1.6 × 10^6^ ± 4.2 × 10^5 (b)^	2.3 × 10^5^ ± 4.7 × 10^4 (a)^	2.3 × 10^5^ ± 5.2 × 10^4 (a)^	1.2 × 10^6^ ± 1.4 × 10^5 (ab)^	1.6 × 10^6^ ± 3.6 × 10^5 (b)^
1-Octen-3-ol	nd	2.1 × 10^6^ ± 2.9 × 10^5 (a)^	nd	1.7 × 10^6^ ± 2.1 × 10^5 (a)^	nd	nd	nd	nd
Phenol	2.5 × 10^6^ ± 1.1 × 10^5 (b)^	3.1 × 10^6^ ± 2.1 × 10^5 (b)^	3.2 × 10^6^ ± 1.8 × 10^5 (b)^	2.5 × 10^6^ ± 1.3 × 10^5 (b)^	2.5 × 10^5^ ± 6.1 × 10^4 (a)^	1.3 × 10^6^ ± 2.4 × 10^5 (ab)^	6.7 × 10^6^ ± 5.4 × 10^5 (c)^	2.2 × 10^6^ ± 3.5 × 10^5 (b)^
6-Methyl-5-hepten-2-ol	1.9 × 10^6^ ± 2.1 × 10^5 (bc)^	1.6 × 10^6^ ± 5.5 × 10^5 (bc)^	2.1 × 10^6^ ± 3.3 × 10^5 (c)^	1.3 × 10^6^ ± 3.4 × 10^5 (bc)^	nd	2.3 × 10^5^ ± 3.4 × 10^4 (a)^	1.2 × 10^5^ ± 1.9 × 10^4 (a)^	5.6 × 10^5^ ± 3.9 × 10^4 (ab)^
Isooctanol	3.1 × 10^6^ ± 2.2 × 10^5 (c)^	2.7 × 10^6^ ± 3.1 × 10^5 (c)^	3.8 × 10^6^ ± 3.7 × 10^5 (c)^	2.2 × 10^6^ ± 1.9 × 10^5 (c)^	3.2 × 10^5^ ± 3.3 × 10^4 (b)^	2.3 × 10^5^ ± 3.5 × 10^4 (ab)^	1.2 × 10^5^ ± 3.7 × 10^4 (a)^	2.7 × 10^6^ ± 4.7 × 10^5 (c)^
2-Ethyl-1-hexanol	5.1 × 10^7^ ± 9.9 × 10^5 (b)^	7.9 × 10^7^ ± 1.3 × 10^6 (c)^	6.4 × 10^7^ ± 3.7 × 10^6 (bc)^	5.9 × 10^7^ ± 3.6 × 10^6 (bc)^	3.6 × 10^5^ ± 3.0 × 10^4 (a)^	4.5 × 10^6^ ± 3.4 × 10^5 (b)^	3.2 × 10^6^ ± 2.5 × 10^5 (ab)^	4.3 × 10^6^ ± 2.9 × 10^5 (ab)^
2-Methylphenol	9.4 × 10^5^ ± 2.4 × 10^4 (c)^	9.9 × 10^5^ ± 5.2 × 10^4 (c)^	5.9 × 10^5^ ± 7.5 × 10^4 (bc)^	7.9 × 10^5^ ± 6.0 × 10^4 (c)^	2.3 × 10^4^ ± 6.9 × 10^3 (a)^	3.4 × 10^5^ ± 4.0 × 10^4 (b)^	1.6 × 10^5^ ± 6.7 × 10^4 (ab)^	6.9 × 10^4^ ± 6.9 × 10^3 (ab)^
1-Octanol	2.5 × 10^6^ ± 8.7 × 10^4 (c)^	1.9 × 10^6^ ± 1.9 × 10^5 (c)^	2.5 × 10^6^ ± 2.0 × 10^5 (c)^	1.7 × 10^6^ ± 2.8 × 10^5 (c)^	5.2 × 10^5^ ± 4.1 × 10^4 (ab)^	5.3 × 10^5^ ± 3.7 × 10^4 (ab)^	2.2 × 10^5^ ± 2.3 × 10^4 (a)^	1.2 × 10^6^ ± 2.0 × 10^4 (bc)^
2-Methoxyphenol	2.8 × 10^6^ ± 9.4 × 10^5 (c)^	3.6 × 10^5^ ± 9.7 × 10^5 (c)^	nd	nd	1.2 × 10^5^ ± 1.0 × 10^4 (a)^	1.7 × 10^6^ ± 9.7 × 10^4 (b)^	1.6 × 10^5^ ± 2.2 × 10^4 (a)^	1.1 × 10^6^ ± 1.6 × 10^5 (abc)^
2-Nonen-1-ol	nd	9.1 × 10^5^ ± 9.5 × 10^3 (c)^	8.7 × 10^5^ ± 2.9 × 10^4 (bc)^	6.2 × 10^5^ ± 2.6 × 10^4 (b)^	nd	2.6 × 10^5^ ± 4.0 × 10^4 (ab)^	1.1 × 10^5^ ± 2.2 × 10^4 (a)^	2.5 × 10^5^ ± 4.5 × 10^4 (ab)^
Phenylethanol	4.3 × 10^6^ ± 3.7 × 10^5 (a)^	5.8 × 10^6^ ± 8.0 × 10^5 (a)^	6.8 × 10^6^ ± 7.1 × 10^5 (a)^	6.6 × 10^6^ ± 6.7 × 10^5 (a)^	4.3 × 10^6^ ± 3.7 × 10^5 (a)^	4.4 × 10^6^ ± 4.0 × 10^5 (a)^	6.7 × 10^7^ 5.8 × 10^6 (c)^	3.4 × 10^7^ ± 5.2 × 10^6 (b)^
Aldehydes								
Pentanal	7.3 × 10^6^ ± 3.6 × 10^5 (bc)^	7.8 × 10^6^ ± 9.9 × 10^5 (c)^	3.2 × 10^6^ ± 1.7 × 10^5 (b)^	3.5 × 10^6^ ± 4.1 × 10^5 (b)^	2.2 × 10^4^ ± 8.1 × 10^2 (a)^	2.3 × 10^5^ ± 6.7 × 10^2 (a)^	2.1 × 10^6^ ± 8.8 × 10^4 (ab)^	2.3 × 10^6^ ± 9.3 × 10^5 (ab)^
2-Pentenal	4.2 × 10^6^ ± 1.4 × 10^5 (b)^	1.0 × 10^7^ ± 9.3 × 10^5 (c)^	4.5 × 10^6^ ± 1.5 × 10^5 (bc)^	2.4 × 10^6^ ± 3.5 × 10^4 (ab)^	2.9 × 10^5^ ± 1.6 × 10^4 (a)^	1.9 × 10^5^ ± 5.9 × 10^3 (a)^	1.7 × 10^6^ ± 1.1 × 10^5 (ab)^	2.9 × 10^6^ ± 8.7 × 10^4 (ab)^
2-Methyl-4-pentenal	2.5 × 10^8^ ± 4.8 × 10^7 (b)^	2.5 × 10^6^ ± 7.0 × 10^5 (a)^	4.0 × 10^6^ ± 9.0 × 10^5 (a)^	1.3 × 10^8^ ± 9.7 × 10^6 (b)^	5.3 × 10^6^ ± 2.6 × 10^5 (a)^	5.6 × 10^7^ ± 1.0 × 10^6 (ab)^	6.7 × 10^7^ ± 1.2 × 10^7 (ab)^	4.6 × 10^7^ ± 8.2 × 10^6 (ab)^
Hexanal	1.8 × 10^8^ ± 1.7 × 10^7 (b)^	1.5 × 10^8^ ± 1.9 × 10^5 (b)^	2.6 × 10^8^ ± 1.8 × 10^7 (b)^	2.3 × 10^8^ ± 7.4 × 10^6 (b)^	7.3 × 10^6^ ± 8.1 × 10^5 (a)^	2.7 × 10^7^ ± 6.7 × 10^6 (a)^	1.9 × 10^7^ ± 6.0 × 10^5 (a)^	1.7 × 10^7^ ± 2.2 × 10^6 (a)^
3-Hexenal	4.5 × 10^8^ ± 1.4 × 10^7 (b)^	4.6 × 10^8^ ± 5.0 × 10^7 (b)^	2.2 × 10^8^ ± 1.3 × 10^7 (ab)^	5.1 × 10^8^ ± 2.2 × 10^7 (b)^	3.3 × 10^7^ ± 3.8 × 10^6 (a)^	3.6 × 10^7^ ± 2.0 × 10^6 (a)^	2.9 × 10^7^ ± 1.6 × 10^6 (a)^	3.9 × 10^7^ ± 2.9 × 10^6 (a)^
2-Hexenal	1.7 × 10^9^ ± 1.0 × 10^8 (c)^	2.5 × 10^9^ ± 2.5 × 10^8 (d)^	2.2 × 10^9^ ± 2.4 × 10^8 (cd)^	2.2 × 10^9^ ± 3.6 × 10^8 (cd)^	1.5 × 10^7^ ± 1.2 × 10^6 (a)^	2.4 × 10^7^ ± 6.7 × 10^6 (a)^	3.3 × 10^7^ ± 4.6 × 10^6 (a)^	9.8 × 10^7^ ± 2.5 × 10^6 (b)^
(Z)-4-Heptenal	7.6 × 10^6^ ± 5.0 × 10^4 (b)^	9.8 × 10^6^ ± 1.5 × 10^5 (c)^	7.7 × 10^6^ ± 2.5 × 10^5 (b)^	8.0 × 10^6^ ± 2.8 × 10^5 (bc)^	4.7 × 10^4^ ± 2.1 × 10^3 (a)^	5.4 × 10^5^ ± 1.1 × 10^4 (ab)^	2.4 × 10^6^ ± 5.0 × 10^5 (a)^	6.5 × 10^6^ ± 9.4 × 10^4 (b)^
(2E,4E)-2,4-Hexadienal	2.6 × 10^7^ ± 1.7 × 10^6 (b)^	1.9 × 10^7^ ± 1.2 × 10^6 (ab)^	3.4 × 10^7^ ± 6.0 × 10^6 (c)^	3.3 × 10^7^ ± 6.8 × 10^6 (c)^	1.4 × 10^7^ ± 4.2 × 10^6 (a)^	2.7 × 10^7^ ± 7.3 × 10^6 (bc)^	1.1 × 10^7^ ± 1.2 × 10^6 (a)^	1.8 × 10^7^ ± 6.0 × 10^6 (a)^
Heptanal	7.6 × 10^6^ ± 2.2 × 10^5 (bc)^	9.7 × 10^6^ ± 2.5 × 10^5 (bc)^	1.1 × 10^7^ ± 1.1 × 10^6 (c)^	8.0 × 10^6^ ± 2.3 × 10^5 (bc)^	2.3 × 10^5^ ± 3.0 × 10^4 (a)^	2.3 × 10^5^ ± 3.6 × 10^4 (a)^	2.6 × 10^5^ ± 1.4 × 10^4 (a)^	1.2 × 10^6^ ± 1.9 × 10^5 b)^
(E)-2-Heptenal	1.6 × 10^6^ ± 1.8 × 10^5 (ab)^	2.4 × 10^6^ ± 1.7 × 10^5 (b)^	1.8 × 10^6^ ± 2.8 × 10^5 (ab)^	1.8 × 10^6^ ± 1.9 × 10^5 (ab)^	1.3 × 10^5^ ± 4.8 × 10^3 (a)^	1.6 × 10^5^ ± 2.2 × 10^4 (a)^	1.2 × 10^5^ ± 2.0 × 10^4 (a)^	1.5 × 10^5^ ± 3.6 × 10^4 (a)^
(E)-4-Oxohex-2-enal	5.4 × 10^7^ ± 3.4 × 10^6 (a)^	2.2 × 10^8^ ± 6.9 × 10^6 (b)^	1.4 × 10^8^ ± 2.2 × 10^7 (ab)^	6.9 × 10^7^ ± 4.3 × 10^6 (a)^	1.8 × 10^8^ ± 1.7 × 10^7 (ab)^	2.1 × 10^8^ ± 1.4 × 10^7 (ab)^	1.9 × 10^8^ ± 4.6 × 10^7 (ab)^	1.3 × 10^8^ ± 6.0 × 10^6 (ab)^
Benzaldehyde	3.4 × 10^7^ ± 5.6 × 10^6 (bc)^	8.2 × 10^7^ ± 4.0 × 10^6 (c)^	1.1 × 10^7^ ± 1.8 × 10^6 (b)^	3.6 × 10^7^ ± 4.1 × 10^6 (bc)^	2.1 × 10^4^ ± 2.3 × 10^3 (a)^	2.4 × 10^4^ ± 9.6 × 10^2 (a)^	2.3 × 10^3^ ± 2.1 × 10^2 (a)^	2.8 × 10^4^ ± 2.7 × 10^3 (a)^
Octanal	nd	2.3 × 10^7^ ± 3.8 × 10^6 ab()^	3.6 × 10^7^ ± 2.6 × 10^6 (b)^	2.7 × 10^7^ ± 6.6 × 10^6 (b)^	2.4 × 10^5^ ± 3.8 × 10^4 (a)^	5.5 × 10^5^ ± 3.5 × 10^4 (a)^	2.1 × 10^6^ ± 2.4 × 10^5 (a)^	3.3 × 10^6^ ± 2.9 × 10^5 (a)^
(Z)-6-Nonenal	nd	2.5 × 10^6^ ± 3.1 × 10^5 (c)^	nd	1.7 × 10^6^ ± 8.5 × 10^4 (c)^	nd	1.6 × 10^5^ ± 1.3 × 10^4 (a)^	nd	2.4 × 10^5^ ± 2.1 × 10^4 (bc)^
(2E,4E)-2,4-Heptadienal	9.9 × 10^6^ ± 2.2 × 10^5 (ab)^	1.3 × 10^7^ ± 1.6 × 10^6 (ab)^	1.3 × 10^7^ ± 3.6 × 10^6 (ab)^	1.4 × 10^7^ ± 1.5 × 10^6 (ab)^	3.7 × 10^6^ ± 1.8 × 10^5 (a)^	2.3 × 10^7^ ± 2.4 × 10^6 (b)^	1.2 × 10^7^ ± 1.4 × 10^6 (ab)^	7.2 × 10^6^ ± 1.5 × 10^5 (ab)^
2-Hydroxybenzaldehyde	3.7 × 10^5^ ± 3.4 × 10^4 (b)^	nd	nd	nd	2.3 × 10^5^ ± 8.1 × 10^3 (ab)^	1.1 × 10^6^ ± 1.9 × 10^5 (b)^	3.3 × 10^4^ ± 5.5 × 10^2 (a)^	1.1 × 10^5^ ± 9.6 × 10^3 (ab)^
2-Octenal	nd	1.6 × 10^6^ ± 2.5 × 10^5 (a)^	nd	2.2 × 10^6^ ± 6.2 × 10^4 (a)^	6.1 × 10^5^ ± 3.0 × 10^4 (a)^	2.1 × 10^7^ ± 3.3 × 10^6 (b)^	6.4 × 10^5^ ± 1.8 × 10^4 (a)^	1.2 × 10^7^ ± 2.3 × 10^6 (b)^
2,6-Dimethyl-5-heptenal	4.0 × 10^6^ ± 3.4 × 10^5 (b)^	3.4 × 10^6^ ± 5.3 × 10^5 (ab)^	3.5 × 10^6^ ± 5.1 × 10^5 (ab)^	3.2 × 10^6^ ± 2.8 × 10^5 (ab)^	nd	nd	nd	1.6 × 10^6^ ± 2.0 × 10^5 (a)^
4-Methylbenzaldehyde	nd	1.2 × 10^6^ ± 2.5 × 10^5 (a)^	nd	nd	6.8 × 10^3^ ± 1.2 × 10^3 (a)^	2.1 × 10^5^ ± 4.1 × 10^4 (ab)^	1.7 × 10^5^ ± 3.2 × 10^4 (ab)^	7.8 × 10^4^ ± 4.2 × 10^3 (ab)^
Nonanal	3.1 × 10^6^ ± 3.1 × 10^5 (ab)^	3.4 × 10^6^ ± 3.2 × 10^5 (b)^	3.1 × 10^6^ ± 2.0 × 10^5 (ab)^	3.0 × 10 ± 2.8 × 10^5 (ab)^	2.2 × 10^6^ ± 2.3 × 10^5 (ab)^	1.2 × 10^6^ ± 2.1 × 10^5 (ab)^	2.3 × 10^5^ ± 6.4 × 10^3 (a)^	1.5 × 10^6^ ± 3.2 × 10^5 (ab)^
(E,Z)-2,6-Nonadienal	2.2 × 10^6^ ± 2.7 × 10^5 (bc)^	3.1 × 10^6^ ± 2.2 × 10^5 (c)^	1.9 × 10^6^ ± 1.9 × 10^5 (bc)^	1.5 × 10^6^ ± 1.7 × 10^5 (bc)^	nd	2.2 × 10^5^ ± 1.8 × 10^4 (a)^	4.4 × 10^5^ ± 5.0 × 10^4 (ab)^	1.6 × 10^5^ ± 2.4 × 10^4 (a)^
4-Ethylbenzaldehyde	3.4 × 10^6^ ± 1.5 × 10^5 (b)^	4.7 × 10^6^ ± 9.4 × 10^5 (b)^	4.3 × 10^6^ ± 1.7 × 10^5 (b)^	4.6 × 10^6^ ± 1.7 × 10^5 (b)^	1.3 × 10^6^ ± 2.2 × 10^5 (a)^	2.3 × 10^6^ ± 2.2 × 10^5 (ab)^	8.0 × 10^5^ ± 5.6 × 10^4 (ab)^	8.0 × 10^5^ ± 6.1 × 10^4 (a)^
3-Thujen-10-al	5.2 × 10^5^ ± 2.6 × 10^4 (a)^	4.2 × 10^6^ ± 2.0 × 10^5 (b)^	nd	nd	4.7 × 10^5^ ± 1.3 × 10^4 (a)^	5.7 × 10^5^ ± 2.3 × 10^4 (a)^	5.1 × 10^5^ ± 1.7 × 10^4 (a)^	1.4 × 10^6^ ± 2.0 × 10^5 (a)^
Decanal	1.1 × 10^6^ ± 1.1 × 10^5 (c)^	6.7 × 10^5^ ± 3.6 × 10^4 (bc)^	9.7 × 10^5^ ± 7.9 × 10^3 (c)^	nd	2.3 × 10^4^ ± 3.7 × 10^3 (a)^	2.2 × 10^5^ ± 5.9 × 10^4 (b)^	2.3 × 10^4^ ± 2.2 × 10^3 (a)^	3.2 × 10^5^ ± 3.1 × 10^4 (b)^
**Ketones**								
1-Penten-3-one	1.7 × 10^7^ ± 1.3 × 10^6 (b)^	1.7 × 10^7^ ± 7.2 × 10^5 (b)^	1.3 × 10^7^ ± 9.5 × 10^5 (b)^	1.7 × 10^7^ ± 2.9 × 10^6 (b)^	2.8 × 10^6^ ± 3.5 × 10^5 (a)^	1.3 × 10^6^ ± 2.0 × 10^5 (ab)^	4.3 × 10^5^ ± 2.8 × 10^4 (a)^	1.5 × 10^6^ ± 2.4 × 10^5 (a)^
3-Pentanone	1.5 × 10^6^ ± 1.2 × 10^5 (ab)^	1.4 × 10^6^ ± 3.8 × 10^5 (ab)^	1.5 × 10^6^ ± 3.7 × 10^5 (ab)^	nd	2.2 × 10^5^ ± 3.5 × 10^4 (a)^	5.9 × 10^6^ ± 5.1 × 10^5 (b)^	nd	nd
5-Methyl-2-hexanone	nd	2.9 × 10^5^ ± 4.9 × 10^3 (abc)^	nd	nd	2.9 × 10^5^ ± 1.7 × 10^4 (ab)^	1.1 × 10^5^ ± 2.3 × 10^4 (abc)^	2.6 × 10^5^ ± 1.4 × 10^4 (b)^	8.2 × 10^5^ ± 3.5 × 10^4 (c)^
5-Ethyl-2(5H)-furanone	nd	nd	7.7 × 10^3^ ± 6.6 × 10^2 (a)^	nd	nd	nd	6.4 × 10^3^ ± 1.8 × 10^2 (a)^	nd
6-Methyl-2-heptanone	nd	nd	nd	7.5 × 10^5^ ± 3.4 × 10^4 (a)^	nd	nd	nd	6.7 × 10^5^ ± 3.6 × 10^4 (a)^
1-Octen-3-one	1.6 × 10^6^ ± 2.3 × 10^5 (bc)^	1.8 × 10^6^ ± 5.0 × 10^5 (bc)^	1.6 × 10^6^ ± 6.0 × 10^5 (c)^	nd	1.6 × 10^5^ ± 4.9 × 10^4 (a)^	2.3 × 10^5^ ± 4.9 × 10^4 (b)^	1.5 × 10^5^ ± 4.2 × 10^4 (ab)^	2.2 × 10^5^ ± 3.5 × 10^4 (abc)^
6-Methyl-5-hepten-2-one	2.4 × 10^6^ ± 2.6 × 10^5 (b)^	2.2 × 10^6^ ± 1.8 × 10^5 (b)^	2.1 × 10^6^ ± 3.3 × 10^5 (b)^	2.1 × 10^5^ ± 3.8 × 10^4 (b)^	2.4 × 10^5^ ± 5.0 × 10^4 (ab)^	2.2 × 10^5^ ± 2.3 × 10^3 (a)^	1.4 × 10^5^ ± 4.9 × 10^3 (a)^	2.3 × 10^6^ ± 3.0 × 10^5 (b)^
2,2,6-Trimethyl-cyclohexanone	8.7 × 10^5^ ± 5.6 × 10^4 (ab)^	1.8 × 10^6^ ± 4.8 × 10^5 (bc)^	nd	1.4 × 10^6^ ± 5.0 × 10^5 (ab)^	9.7 × 10^5^ ± 1.6 × 10^4 (ab)^	1.0 × 10^5^ ± 4.5 × 10^3 (a)^	2.3 × 10^6^ ± 2.3 × 10^5 (c)^	2.4 × 10^6^ ± 2.7 × 10^5 (c)^
Acetophenone	4.2 × 10^6^ ± 2.2 × 10^5 (b)^	1.3 × 10^6^ ± 3.5 × 10^5 (ab)^	1.5 × 10^6^ ± 3.8 × 10^5 (ab)^	2.4 × 10^6^ ± 2.8 × 10^5 (ab)^	1.3 × 10^6^ ± 3.7 × 10^5 (a)^	4.7 × 10^6^ ± 5.7 × 10^5 (b)^	8.0 × 10^6^ ± 1.0 × 10^6 (bc)^	1.3 × 10^7^ ± 6.5 × 10^5 (c)^
Ketoisophorone	nd	nd	nd	6.2 × 10^5^ ± 3.6 × 10^4 (a)^	4.7 × 10^5^ ± 7.0 × 10^4 (b)^	4.6 × 10^5^ ± 7.1 × 10^4 (b)^	4.2 × 10^5^ ± 4.2 × 10^4 (b)^	4.3 × 10^5^ ± 3.7 × 10^4 (b)^
2-Hydroxyacetophenone	6.0 × 10^5^ ± 3.4 × 10^4 (a)^	3.3 × 10^6^ ± 1.7 × 10^5 (a)^	3.1 × 10^6^ ± 1.4 × 10^5 (ab)^	3.4 × 10^6^ ± 4.5 × 10^5 (ab)^	1.7 × 10^5^ ± 1.9 × 10^4 (a)^	6.6 × 10^7^ ± 1.2 × 10^6 (b)^	3.2 × 10^6^ ± 2.0 × 10^5 (a)^	6.5 × 10^7^ ± 9.9 × 10^5 (b)^
3,6,6-Trimethyl-2-cyclohexen-1-one	3.9 × 10^5^ ± 4.0 × 10^4 (a)^	7.2 × 10^5^ ± 3.8 × 10^4 (a)^	3.3 × 10^5^ ± 4.4 × 10^4 (a)^	3.9 × 10^5^ ± 6.2 × 10^4 (a)^	8.9 × 10^5^ ± 8.0 × 10^4 (a)^	9.2 × 10^5^ ± 2.3 × 10^4 (a)^	4.7 × 10^6^ ± 2.3 × 10^5 (a)^	6.9 × 10^7^ ± 2.5 × 10^6 (b)^
Esters								
Methyl acetate	1.5 × 10^6^ ± 2.0 × 10^5 (bc)^	8.3 × 10^5^ ± 6.0 × 10^4 (b)^	1.5 × 10^6^ ± 4.1 × 10^5 (bc)^	4.7 × 10^5^ ± 3.0 × 10^4 (ab)^	1.0 × 10^4^ ± 3.0 × 10^2 (a)^	1.8 × 10^6^ ± 3.5 × 10^5 (c)^	7.1 × 10^5^ ± 3.9 × 10^4 (ab)^	4.3 × 10^5^ ± 3.2 × 10^4 (ab)^
Ethyl acetate	2.0 × 10^7^ ± 4.8 × 10^5 (c)^	5.1 × 10^6^ ± 3.0 × 10^5 (b)^	5.1 × 10^6^ ± 4.7 × 10^5 (b)^	4.9 × 10^6^ ± 2.9 × 10^5 (b)^	1.9 × 10^6^ ± 2.4 × 10^5 (a)^	2.1 × 10^6^ ± 4.2 × 10^5 (a)^	1.5 × 10^6^ ± 6.6 × 10^5 (a)^	3.6 × 10^6^ ± 5.6 × 10^5 (b)^
Butyl acetate	6.5 × 10^6^ ± 6.9 × 10^5 (c)^	2.2 × 10^6^ ± 2.8 × 10^5 (b)^	8.3 × 10^5^ ± 7.3 × 10^4 (a)^	1.1 × 10^6^ ± 2.2 × 10^5 (ab)^	7.4 × 10^5^ ± 3.9 × 10^4 (a)^	2.3 × 10^6^ ± 3.3 × 10^5 (b)^	6.9 × 10^5^ ± 7.0 × 10^4 (a)^	6.5 × 10^6^ ± 4.1 × 10^5 (c)^
Hexyl acetate	1.5 × 10^7^ ± 2.3 × 10^6 (bc)^	5.7 × 10^6^ ± 3.8 × 10^5 (b)^	1.1 × 10^7^ ± 8.2 × 10^5 (bc)^	5.3 × 10^6^ ± 2.8 × 10^5 (bc)^	1.3 × 10^6^ ± 2.0 × 10^5 (a)^	3.6 × 10^6^ ± 3.6 × 10^5 (ab)^	3.9 × 10^6^ ± 5.5 × 10^5 (ab)^	2.6 × 10^7^ ± 2.2 × 10^6 (c)^
Butyl hexanoate	5.1 × 10^6^ ± 2.8 × 10^5 (b)^	nd	8.3 × 10^5^ ± 7.3 × 10^4 (a)^	nd	nd	nd	nd	nd
Methyl salicylate	1.3 × 10^4^ ± 2.1 × 10^3 (a)^	1.3 × 10^5^ ± 4.9 × 10^4 (bc)^	5.8 × 10^4^ ± 3.3 × 10^3 (ab)^	1.3 × 10^5^ ± 2.0 × 10^4 ()bc^	1.1 × 10^4^ ± 1.8 × 10^3 (a)^	3.9 × 10^5^ ± 4.8 × 10^4 (c)^	3.1 × 10^5^ ± 2.3 × 10^4 (bc)^	4.5 × 10^5^ ± 2.5 × 10^4 (c)^
Ethyl salicylate	5.1 × 10^5^ ± 2.1 × 10^4 (a)^	8.2 × 10^5^ ± 1.8 × 10^4 (ab)^	6.1 × 10^5^ ± 7.6 × 10^4 (a)^	5.1 × 10^5^ ± 1.4 × 10^4 (a)^	6.0 × 10^6^ ± 1.4 × 10^5 (b)^	7.6 × 10^6^ ± 6.9 × 10^5 (bc)^	2.3 × 10^6^ ± 2.5 × 10^5 (b)^	4.4 × 10^7^ ± 2.5 × 10^6 (c)^
Isoamyl salicylate	nd	nd	nd	nd	nd	1.1 × 10^4^ ± 8.7 × 10^2 (a)^	nd	2.4 × 10^5^ ± 2.7 × 10^4 (a)^
Terpenoids								
α-Pinene	4.6 × 10^5^ ± 6.4 × 10^4 (b)^	2.8 × 10^5^ ± 6.4 × 10^4 (a)^	3.7 × 10^5^ ± 4.3 × 10^4 (ab)^	2.2 × 10^5^ ± 1.4 × 10^4 (a)^	6.4 × 10^6^ ± 2.6 × 10^5 (c)^	6.2 × 10^6^ ± 2.7 × 10^5 (c)^	3.3 × 10^6^ ± 1.8 × 10^5 (ab)^	3.5 × 10^7^ ± 3.9 × 10^6 (abc)^
(-)-Sabinene	2.6 × 10^8^ ± 3.2 × 10^7 (b)^	2.9 × 10^6^ ± 6.1 × 10^5 (a)^	nd	nd	4.6 × 10^5^ ± 4.5 × 10^4 (a)^	6.3 × 10^6^ ± 2.6 × 10^5 (a)^	nd	nd
β-Pinene	2.9 × 10^6^ ± 5.3 × 10^5 (c)^	nd	3.5 × 10^6^ ± 3.0 × 10^5 (c)^	2.4 × 10^6^ ± 1.3 × 10^5 (c)^	5.5 × 10^3^ ± 6.9 × 10^1 (a)^	1.3 × 10^5^ ± 2.6 × 10^4 (b)^	1.3 × 10^5^ ± 1.1 × 10^4 (b)^	1.3 × 10^5^ ± 2.1 × 10^4 (b)^
β-Myrcene	1.6 × 10^7^ ± 6.5 × 10^6 (a)^	2.7 × 10^7^ ± 5.9 × 10^6 (ab)^	1.6 × 10^7^ ± 6.2 × 10^6 (a)^	2.0 × 10^7^ 4.7 × 10^6 (ab)^	4.7 × 10^5^ ± 6.3 × 10^4 (a)^	1.7 × 10^8^ ± 4.9 × 10^7 (c)^	3.2 × 10^7^ ± 4.2 × 10^6 (ab)^	7.3 × 10^7^ ± 4.5 × 10^6 (bc)^
α-Phellandrene	3.2 × 10^7^ ± 1.6 × 10^6 (c)^	1.3 × 10^7^ ± 1.4 × 10^6 (bc)^	1.9 × 10^7^ ± 4.0 × 10^6 (c)^	2.4 × 10^6^ ± 1.6 × 10^5 (a)^	3.4 × 10^7^ ± 8.9 × 10^5 (c)^	2.6 × 10^7^ ± 2.1 × 10^6 (b)^	1.3 × 10^7^ ± 1.7 × 10^6 (abc)^	1.0 × 10^7^ ± 8.1 × 10^5 (ab)^
2-Carene	1.7 × 10^8^ ± 9.2 × 10^6 (b)^	9.5 × 10^7^ ± 6.7 × 10^5 (b)^	1.4 × 10^8^ ± 3.4 × 10^7 (b)^	9.3 × 10^7^ ± 8.9 × 10^6 (ab)^	4.7 × 10^6^ ± 7.8 × 10^4 (a)^	4.7 × 10^4^ ± 1.4 × 10^3 (a)^	1.2 × 10^7^ ± 6.2 × 10^5 (ab)^	2.3 × 10^8^ ± 4.1 × 10^7 (b)^
α-Terpinene	7.3 × 10^5^ ± 1.9 × 10^4 (ab)^	1.3 × 10^5^ ± 1.1 × 10^4 (a)^	1.1 × 10^7^ ± 9.2 × 10^5 (bc)^	3.1 × 10^6^ ± 1.9 × 10^5 (b)^	3.6 × 10^6^ ± 3.0 × 10^5 (b)^	2.2 × 10^7^ ± 1.8 × 10^6 (c)^	1.1 × 10^7^ ± 1.7 × 10^6 (b)^	1.7 × 10^7^ ± 2.5 × 10^6 (bc)^
Limonene	2.1 × 10^8^ ± 2.3 × 10^7 (b)^	1.5 × 10^8^ ± 7.9 × 10^6 (ab)^	1.9 × 10^8^ ± 1.9 × 10^7 (b)^	1.6 × 10^8^ ± 3.2 × 10^7 (ab)^	1.2 × 10^5^ ± 2.2 × 10^4 (a)^	2.3 × 10^4^ ± 3.1 × 10^3 (a)^	1.4 × 10^7^ ± 2.8 × 10^6 (a)^	1.2 × 10^7^ ± 6.3 × 10^5 (a)^
o-Cymene	5.1 × 10^7^ ± 2.4 × 10^6 (c)^	2.6 × 10^7^ ± 6.3 × 10^6 (bc)^	5.1 × 10^7^ ± 4.9 × 10^6 (c)^	3.5 × 10^7^ ± 5.1 × 10^6 (bc)^	4.6 × 10^5^ ± 2.0 × 10^4 (a)^	4.6 × 10^5^ ± 4.9 × 10^4 (a)^	3.9 × 10^6^ ± 6.2 × 10^5 (b)^	2.7 × 10^6^ ± 6.7 × 10^5 (ab)^
p-Cymene	nd	nd	nd	7.5 × 10^5^ ± 3.4 × 10^4 (a)^	nd	nd	4.6 × 10^5^ ± 1.7 × 10^4 (a)^	7.9 × 10^5^ ± 1.7 × 10^4 (a)^
m-Cymene	nd	nd	7.1 × 10^5^ ± 1.7 × 10^4 (a)^	4.9 × 10^5^ ± 4.0 × 10^4 (a)^	nd	nd	4.6 × 10^5^ ± 5.0 × 10^4 (a)^	5.7 × 10^5^ ± 5.6 × 10^4 (a)^
β-Phellandrene	3.7 × 10^8^ ± 5.9 × 10^7 (ab)^	2.3 × 10^8^ ± 1.3 × 10^7 (ab)^	2.9 × 10^8^ ± 2.1 × 10^7 (a)^	1.2 × 10^8^ ± 1.5 × 10^7 (ab)^	9.7 × 10^8^ ± 1.9 × 10^7 (c)^	6.3 × 10^8^ ± 3.4 × 10^7 (bc)^	2.5 × 10^8^ ± 2.4 × 10^7 (ab)^	3.3 × 10^8^ ± 2.4 × 10^7 (abc)^
β-Ocimene	8.1 × 10^5^ ± 3.9 × 10^4 (b)^	7.1 × 10^5^ ± 3.3 × 10^4 (b)^	1.7 × 10^6^ ± 1.9 × 10^5 (c)^	nd	2.5 × 10^5^ ± 2.0 × 10^4 (a)^	4.6 × 10^5^ ± 2.1 × 10^4 (ab)^	1.3 × 10^6^ ± 2.3 × 10^5 (bc)^	nd
α-Ocimene	nd	nd	nd	4.9 × 10^6^ ± 4.9 × 10^5 (b)^	nd	nd	nd	4.6 × 10^5^ ± 1.8 × 10^4 (a)^
γ-Terpinene	9.4 × 10^6^ ± 3.7 × 10^5 (c)^	5.7 × 10^6^ ± 2.2 × 10^5 (bc)^	9.4 × 10^6^ ± 5.2 × 10^5 (c)^	6.0 × 10^6^ ± 4.2 × 10^5 (b)^	5.4 × 10^6^ ± 2.7 × 10^5 (bc)^	3.6 × 10^6^ ± 1.4 × 10^5 (b)^	4.9 × 10^5^ ± 2.0 × 10^4 (a)^	1.8 × 10^6^ ± 1.6 × 10^5 (ab)^
Cryptone	1.2 × 10^6^ ± 3.5 × 10^5 (a)^	nd	6.1 × 10^5^ ± 1.3 × 10^4 (a)^	nd	nd	nd	nd	2.4 × 10^4^ ± 2.1 × 10^3 (a)^
Terpinolene	1.6 × 10^6^ ± 4.3 × 10^5 (a)^	1.3 × 10^6^ ± 2.4 × 10^5 (a)^	2.9 × 10^6^ ± 3.2 × 10^5 (a)^	1.3 × 10^6^ ± 1.9 × 10^5 (a)^	4.6 × 10^4^ ± 3.2 × 10^3 (a)^	4.6 × 10^6^ ± 6.1 × 10^5 (a)^	3.2 × 10^5^ ± 2.4 × 10^4 (a)^	2.3 × 10^7^ ± 5.8 × 10^6 (b)^
β-Cyclocitral	7.8 × 10^5^ ± 8.8 × 10^4 (b)^	9.9 × 10^5^ ± 5.9 × 10^4 (bc)^	1.0 × 10^6^ ± 9.6 × 10^4 (bc)^	8.2 × 10^5^ ± 1.3 × 10^5 (b)^	8.9 × 10^5^ ± 2.9 × 10^5 (b)^	1.7 × 10^6^ ± 8.3 × 10^5 (c)^	1.2 × 10^5^ ± 2.0 × 10^5 (a)^	1.4 × 10^6^ ± 7.0 × 10^5 (bc)^
δ-Elemene	6.9 × 10^5^ ± 3.3 × 10^4 (a)^	1.2 × 10^6^ ± 2.9 × 10^5 (a)^	nd	1.4 × 10^6^ ± 1.3 × 10^5 (a)^	1.3 × 10^4^ ± 7.3 × 10^2 (a)^	1.7 × 10^5^ ± 4.3 × 10^4 (a)^	nd	3.5 × 10^7^ ± 4.7 × 10^6 (b)^
α-Copaene	nd	1.2 × 10^6^ ± 2.6 × 10^5 (c)^	nd	nd	2.2 × 10^5^ ± 1.7 × 10^4 (a)^	6.6 × 10^5^ ± 1.0 × 10^4 (bc)^	4.7 × 10^5^ ± 6.4 × 10^4 (b)^	5.6 × 10^5^ ± 5.7 × 10^4 (bc)^
Isocaryophyllene	1.7 × 10^7^ ± 1.7 × 10^6 (b)^	2.1 × 10^6^ ± 1.6 × 10^5 (a)^	4.4 × 10^6^ ± 2.8 × 10^5 (a)^	5.8 × 10^6^ ± 3.9 × 10^5 (a)^	3.2 × 10^6^ ± 3.4 × 10^5 (a)^	5.4 × 10^6^ ± 1.4 × 10^5 (a)^	2.1 × 10^6^ ± 1.8 × 10^5 (a)^	4.0 × 10^7^ ± 2.6 × 10^6 (b)^
α-Caryophyllene	3.9 × 10^6^ ± 6.7 × 10^5 (ab)^	8.8 × 10^6^ ± 4.9 × 10^5 (b)^	4.5 × 10^6^ ± 3.7 × 10^5 (ab)^	8.4 × 10^6^ ± 1.7 × 10^5 (b)^	nd	9.4 × 10^5^ ± 9.2 × 10^4 (a)^	nd	nd
β-lonone	1.0 × 10^6^ ± 1.7 × 10^5 (a)^	1.1 × 10^6^ ± 9.9 × 10^4 (a)^	1.3 × 10^6^ ± 2.4 × 10^5 (a)^	1.2 × 10^6^ ± 7.2 × 10^4 (a)^	4.7 × 10^5^ ± 4.6 × 10^4 (a)^	4.7 × 10^5^ ± 4.4 × 10^4 (a)^	1.3 × 10^6^ ± 4.9 × 10^5 (a)^	7.9 × 10^6^ ± 3.3 × 10^5 (b)^
Other VOCs								
Acetic acid	nd	nd	nd	nd	nd	nd	7.3 × 10^5^ ± 3.9 × 10^4 (a)^	2.3 × 10^6^ ± 3.9 × 10^5 (a)^
Hexane	3.5 × 10^6^ ± 1.7 × 10^5 (c)^	8.4 × 10^5^ ± 3.8 × 10^4 (b)^	2.0 × 10^5^ ± 2.9 × 10^4 (a)^	1.2 × 10^6^ ± 1.4 × 10^5 (b)^	nd	nd	nd	3.0 × 10^5^ ± 2.1 × 10^4 (a)^
cis-1,2-Dimethyl-cyclopentane	nd	nd	nd	6.0 × 10^6^ ± 7.3 × 10^5 (bc)^	1.3 × 10^6^ ± 3.4 × 10^5 (ab)^	2.3 × 10^6^ ± 3.8 × 10^5 (b)^	3.3 × 10^5^ ± 4.7 × 10^4 (a)^	8.4 × 10^6^ ± 5.2 × 10^5 (c)^
Naphthalene	8.9 × 10^5^ ± 5.1 × 10^4 (b)^	nd	9.7 × 10^5^ ± 4.5 × 10^4 (b)^	8.9 × 10^5^ ± 4.2 × 10^4 (b)^	nd	nd	2.2 × 10^5^ ± 2.4 × 10^4 (a)^	6.4 × 10^5^ ± 5.2 × 10^4 (ab)^
Azulene	Nd	2.0 × 10^6^ ± 5.4 × 10^5 (b)^	nd	nd	nd	1.6 × 10^5^ ± 6.4 × 10^4 (a)^	nd	1.4 × 10^5^ ± 4.8 × 10^4 (a)^
2-Methyltetrahydrofuran	nd	1.0 × 10^6^ ± 5.4 × 10^5 (b)^	nd	nd	nd	1.2 × 10^5^ ± 2.5 × 10^4 (a)^	nd	1.3 × 10^5^ ± 4.2 × 10^4 (a)^
2-Ethylfuran	7.2 × 10^6^ ± 3.7 × 10^5 (b)^	8.9 × 10^6^ ± 8.9 × 10^5 (bc)^	1.4 × 10^7^ ± 4.1 × 10^6 (c)^	9.9 × 10^6^ ± 4.4 × 10^5 (bc)^	1.2 × 10^6^ ± 3.0 × 10^5 (a)^	7.8 × 10^4^ ± 1.2 × 10^4 (a)^	3.3 × 10^5^ ± 1.8 × 10^4 (a)^	3.5 × 10^6^ ± 6.6 × 10^5 (b)^
2-Pentylfuran	1.8 × 10^6^ ± 4.3 × 10^5 (bc)^	2.9 × 10^6^ ± 7.1 × 10^5 (c)^	1.7 × 10^6^ ± 2.1 × 10^5 (bc)^	2.3 × 10^6^ ± 3.7 × 10^5 (bc)^	1.2 × 10^5^ ± 2.4 × 10^4 (a)^	1.6 × 10^6^ ± 2.6 × 10^5 (b)^	1.1 × 10^6^ ± 8.1 × 10^4 (ab)^	1.7 × 10^6^ ± 1.7 × 10^5 (b)^
2-Ethylthiophene	1.2 × 10^8^ ± 2.6 × 10^7 (a)^	1.1 × 10^8^ ± 9.3 × 10^6 (a)^	nd	1.4 × 10^8^ ± 3.5 × 10^7 (a)^	nd	nd	nd	nd
Benzofuran	1.8 × 10^6^ ± 1.5 × 10^5 (b)^	2.6 × 10^6^ ± 4.9 × 10^5 (b)^	1.7 × 10^6^ ± 7.3 × 10^5 (b)^	1.7 × 10^6^ ± 4.5 × 10^5 (b)^	1.4 × 10^5^ ± 8.4 × 10^3 (a)^	1.2 × 10^6^ ± 3.5 × 10^5 (b)^	2.1 × 10^6^ ± 1.9 × 10^5 (b)^	3.2 × 10^5^ ± 3.5 × 10^4 (ab)^

## Data Availability

Data are contained within the article and Supplementary Materials.

## Supplementary Materials

The following supporting information can be downloaded at https://www.mdpi.com/article/10.3390/cells12091271/s1, Figure S1: Representative Total Ion Chromatograms (TICs) of volatile profiles of *B. cinerea* present on the tomato leaves; [1] Ethanol, [2] Hexane, [3] Ethyl acetate, [4] 1-Octen-3-ol (A), and T. virens TRS 106 present in the tomato rhizosphere; [1] 3-Heptanone, [2] Octan-3-one, [3] 3-Octanol, [4] 2,6-Dimethylnonane, [5] 2-Ethyl-1-hexanol, [6] Unknown, [7] Dihydromyrcenol, [8] Viridiflorene, [9] Ledol, [10] Farnesol (B). Figure S2: Representative TICs of VOCs released by tomato leaves; 1D view (A) and representative TICs of VOCs released by tomato leaves; 2D view (B). Figure S3: Representative mass spectra for exemplary VOCs; Hexanal (A), α-Phellandrene (B), 2-Methylphenol (C), 2-Hexenal (D), 3-Hexenal (E), 2-Hydroxyacetophenone (F), 3,3,5-Trimethy-2-cyclohexen-1-one (G), 2-Carene (H), Isocaryophyllene (I), Limonene (J), Myrcene (K), Phenylethanol (L). Table S1: Selected VOCs annotated and putatively annotated in the tomato leaves. Abbreviations: VOC, volatile organic compound, RIexp, relative retention indices calculated against n-alkanes (C_8_–C_20_) on BPX-5 column; RIlit, relative retention indices on non-polar column (BPX-5 or similar stationary phase) reported in the literature (webbook.nist.gov); MS, NIST and Wiley libraries spectra; S, internal standard. Table S2: Volatile metabolites annotated and putatively annotated in the volatile profiles of *B. cinerea* present on the tomato leaves (A) and *T. virens* TRS 106 present in the tomato rhizosphere (B). Abbreviations: VOC, volatile organic compound, RIexp, relative retention indices calculated against n-alkanes (C_8_–C_20_) on BPX-5 column; RIlit, relative retention indices on non-polar column (BPX-5 or similar stationary phase) reported in literature (webbook.nist.gov); MS, NIST and Wiley libraries spectra; S, internal standard.
