# Supplementary material for: Toward the Analysis of Volatile Organic Compounds from Tomato Plants (Solanum lycopersicum L.) Treated with Trichoderma virens or/and Botrytis cinerea
Source: Cells. 2023 Apr 27;12(9):1271. doi: 10.3390/cells12091271 (PMC10177525; doi:10.3390/cells12091271)
Supplement: Supplementary file 1 [file cells-12-01271-s001.zip › cells-2203451-supplementary.pdf]

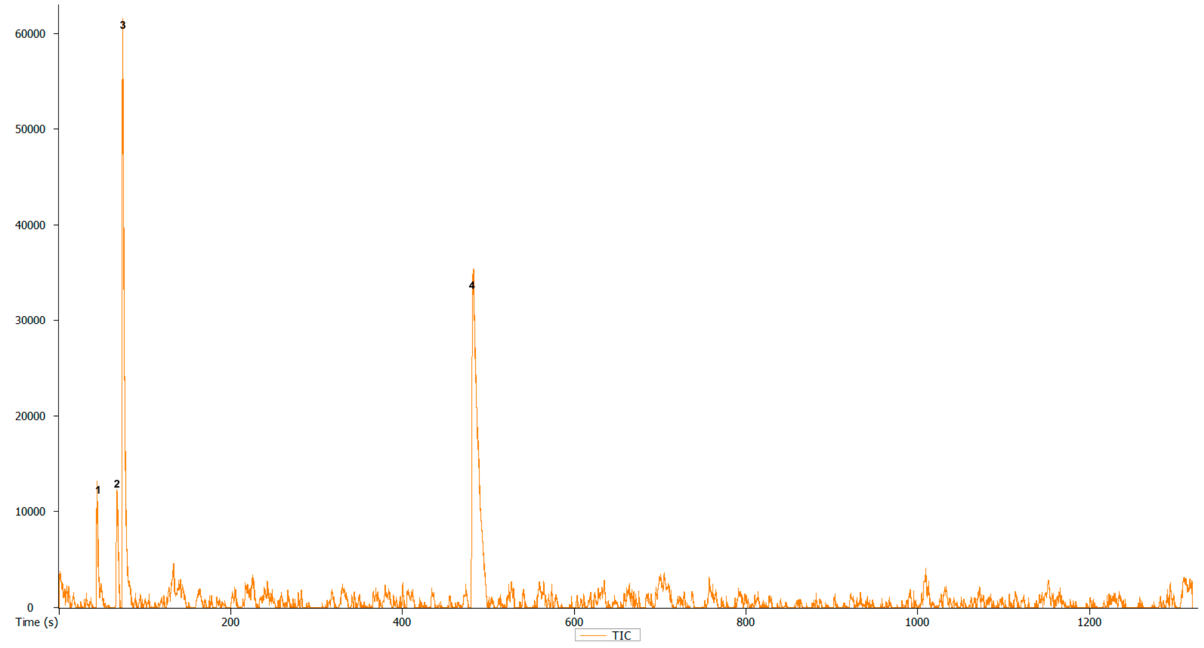

(A)

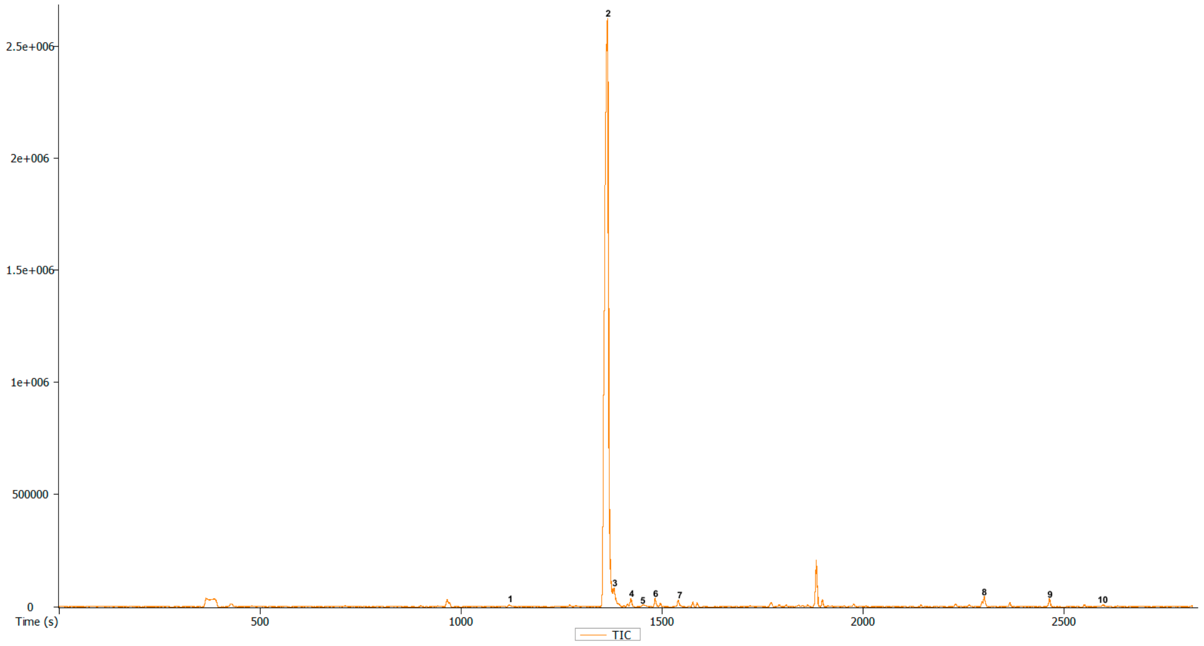

(B)

Figure S1. Representative Total Ion Chromatograms (TICs) of volatile profiles of *B. cinerea* present on the tomato leaves; [1] Ethanol, [2] Hexane, [3] Ethyl acetate, [4] 1-Octen-3-ol **(A),** and *T. virens* TRS 106 present in the tomato rhizosphere; [1] 3-Heptanone, [2] Octan-3-one, [3] 3-Octanol, [4] 2,6-Dimethylnonane, [5] 2-Ethyl-1-hexanol, [6] Unknown, [7] Dihydromyrcenol, [8] Viridiflorene, [9] Ledol, [10] Farnesol **(B)**.

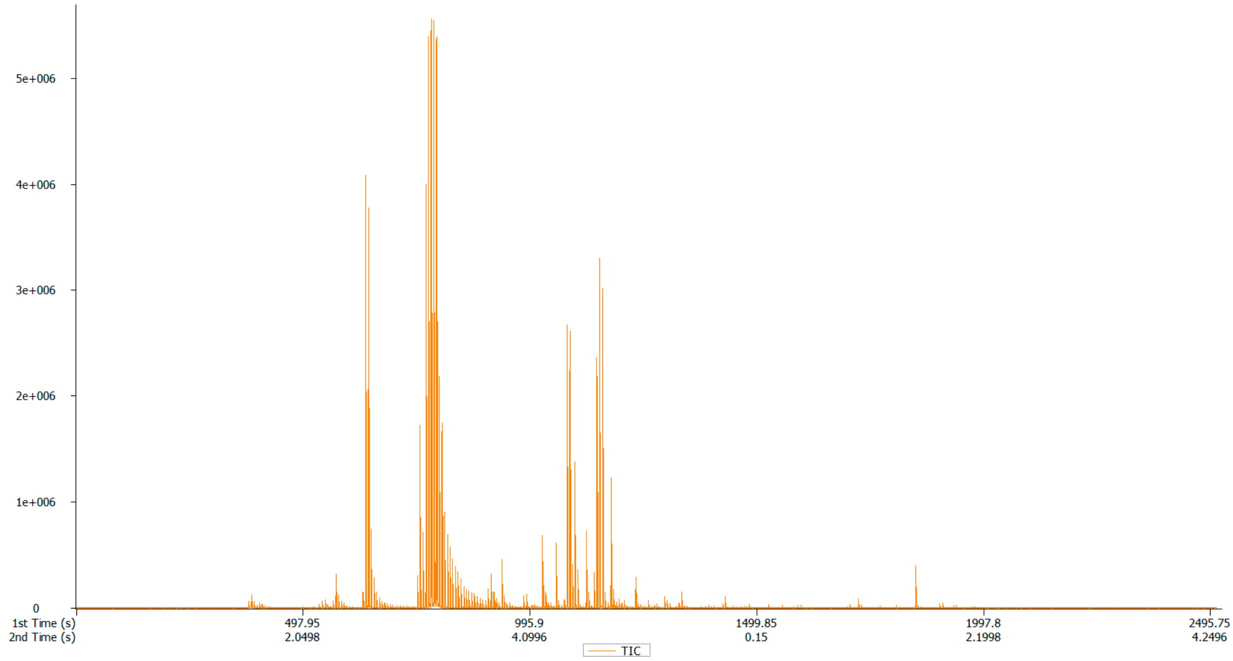

(A)

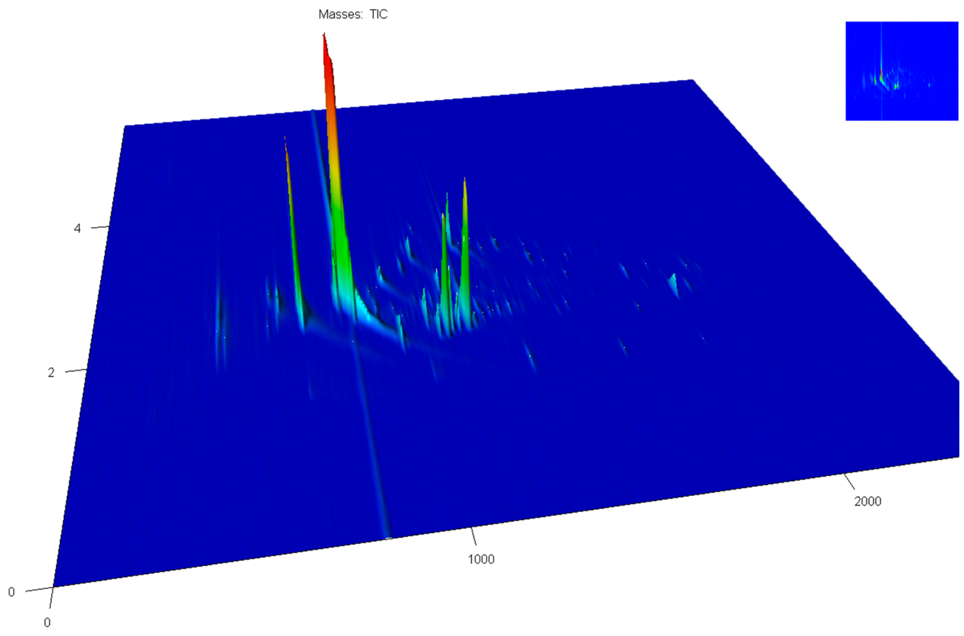

(B)

Figure S2. Representative TICs of VOCs released by tomato leaves; 1D view **(A)** and representative TICs of VOCs released by tomato leaves; 2D view **(B)**.

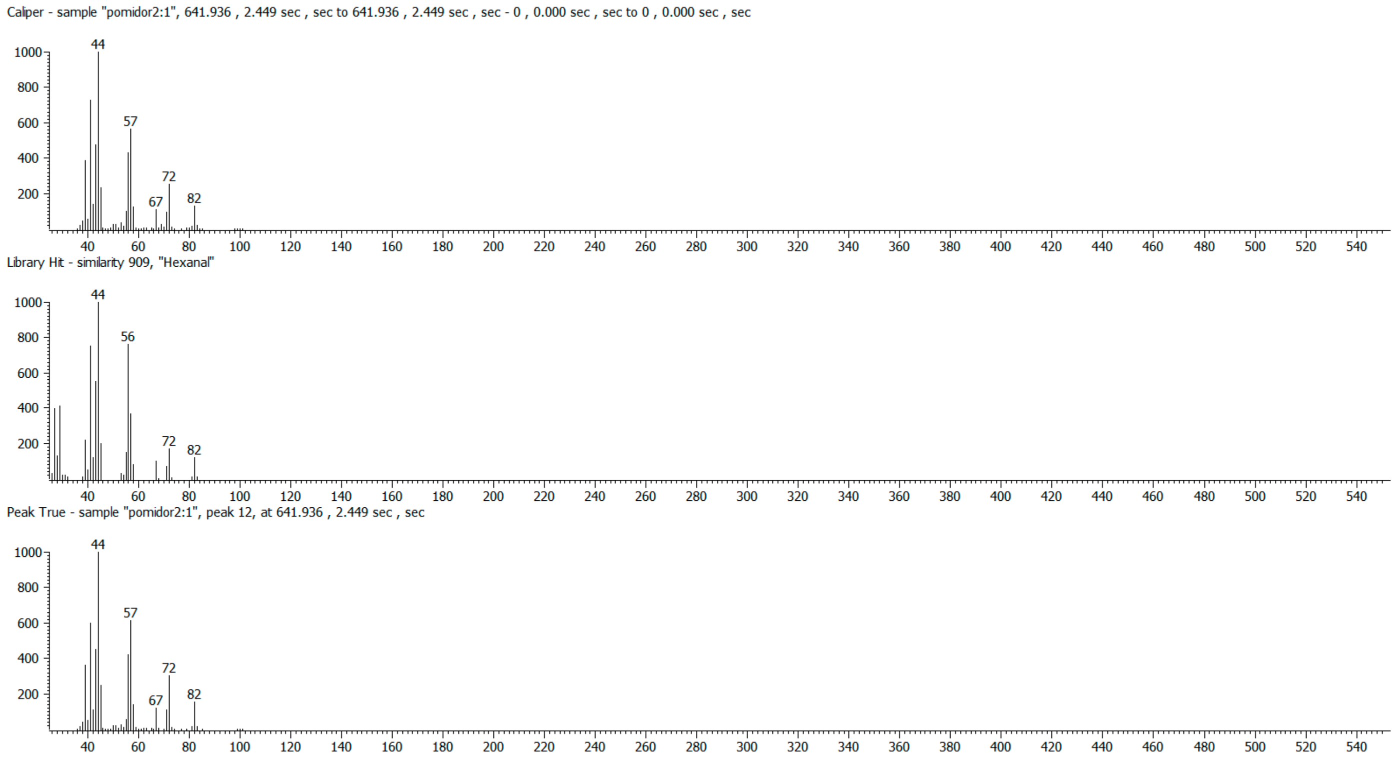
Caliper - sample "pomidor2:1", 641.936 , 2.449 sec , sec to 641.936 , 2.449 sec , sec - 0 , 0.000 sec , sec to 0 , 0.000 sec , sec
44
57
72
67
82
Library Hit - similarity 909, "Hexanal"
44
56
72
82
Peak True - sample "pomidor2:1", peak 12, at 641.936 , 2.449 sec , sec
44
57
72
67
82

(A)

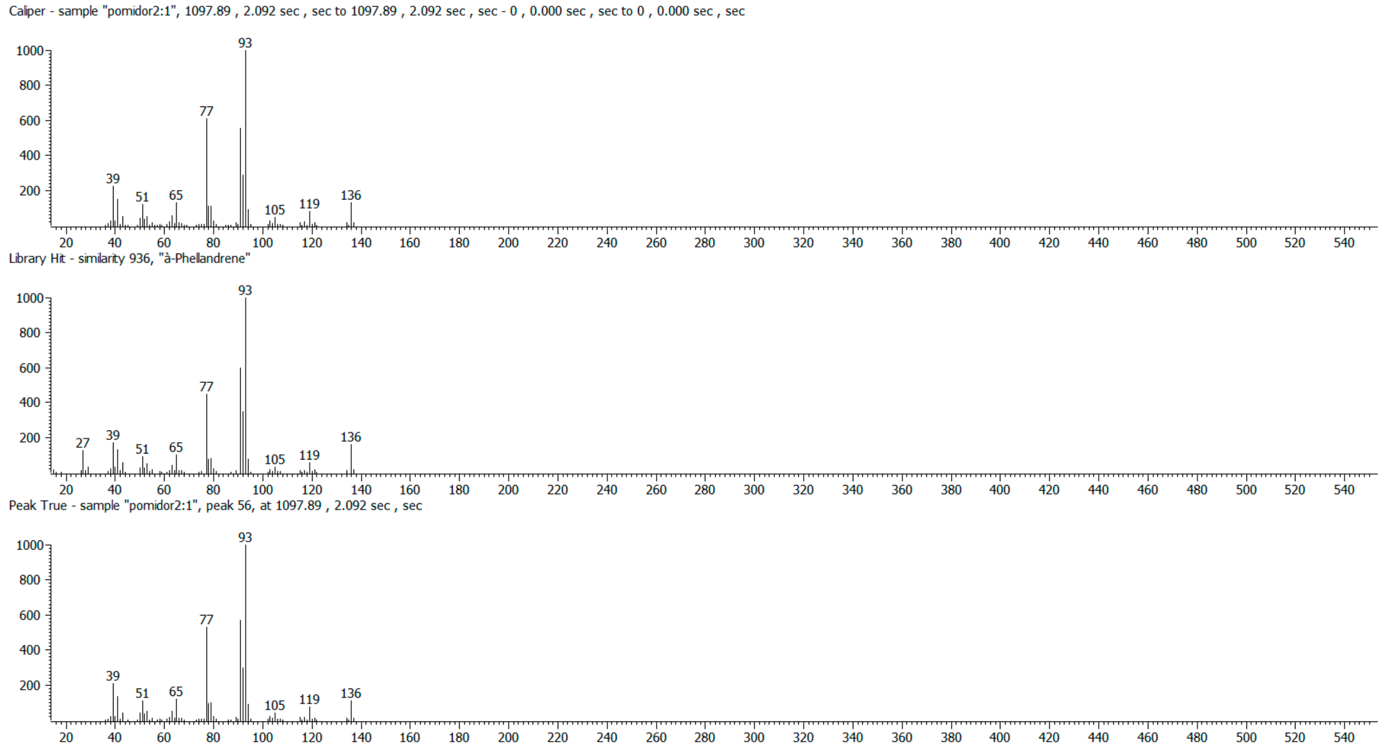
Caliper - sample "pomidor2:1", 1097.89 , 2.092 sec , sec to 1097.89 , 2.092 sec , sec - 0 , 0.000 sec , sec to 0 , 0.000 sec , sec
93
77
39
51
65
105
119
136
Library Hit - similarity 936, "à-Phellandrene"
93
77
27
39
51
65
105
119
136
Peak True - sample "pomidor2:1", peak 56, at 1097.89 , 2.092 sec , sec
93
77
39
51
65
105
119
136

(B)

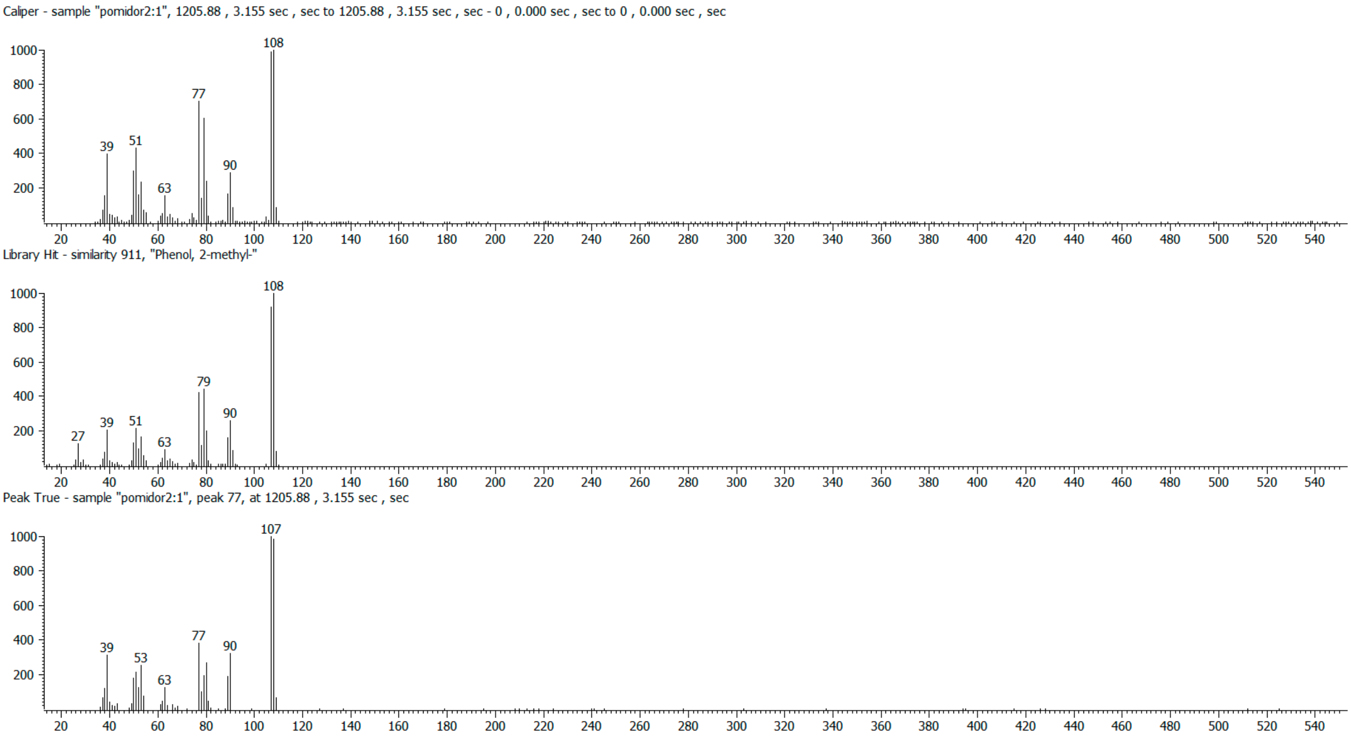
Caliper - sample "pomidor2:1", 1205.88 , 3.155 sec , sec to 1205.88 , 3.155 sec , sec - 0 , 0.000 sec , sec to 0 , 0.000 sec , sec
Library Hit - similarity 911, "Phenol, 2-methyl-"
Peak True - sample "pomidor2:1", peak 77, at 1205.88 , 3.155 sec , sec

(C)

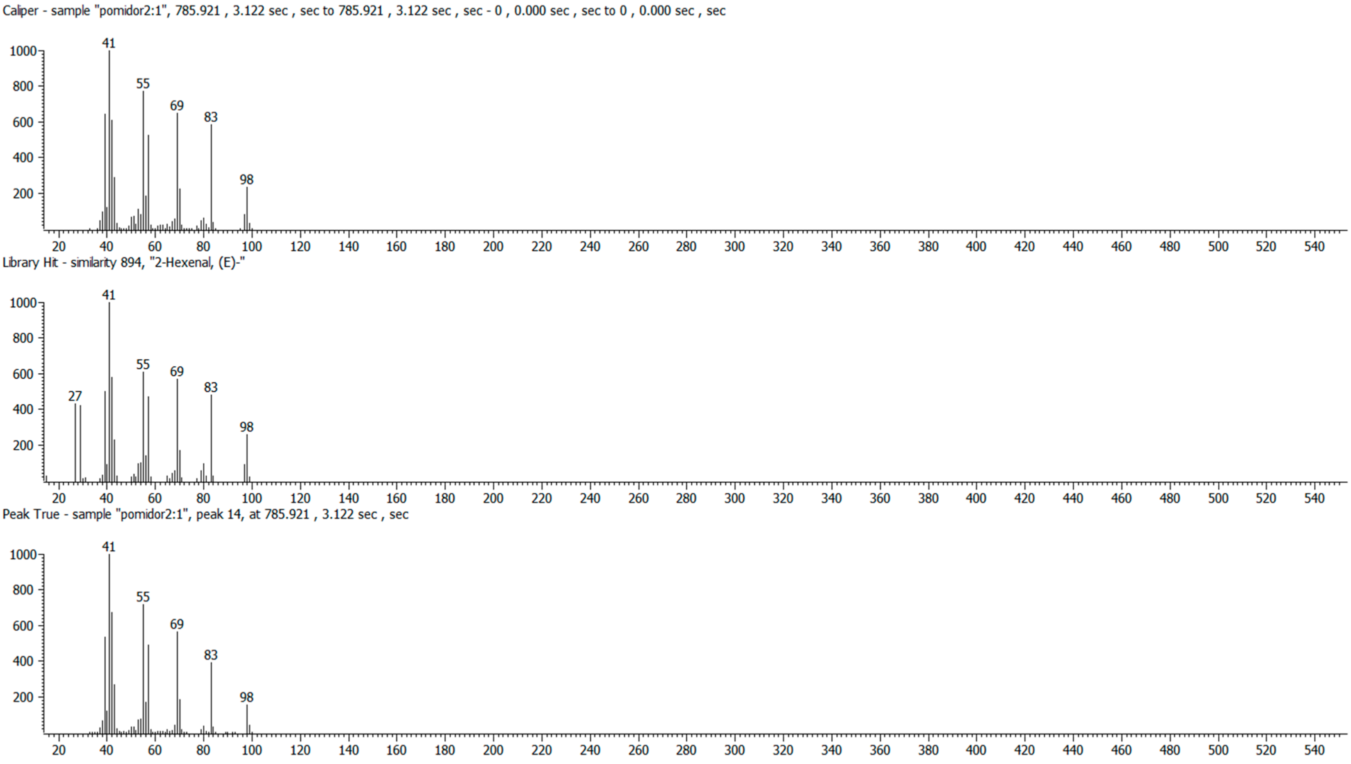
Caliper - sample "pomidor2:1", 785.921 , 3.122 sec , sec to 785.921 , 3.122 sec , sec - 0 , 0.000 sec , sec to 0 , 0.000 sec , sec
Library Hit - similarity 894, "2-Hexenal, (E)-"
Peak True - sample "pomidor2:1", peak 14, at 785.921 , 3.122 sec , sec

(D)

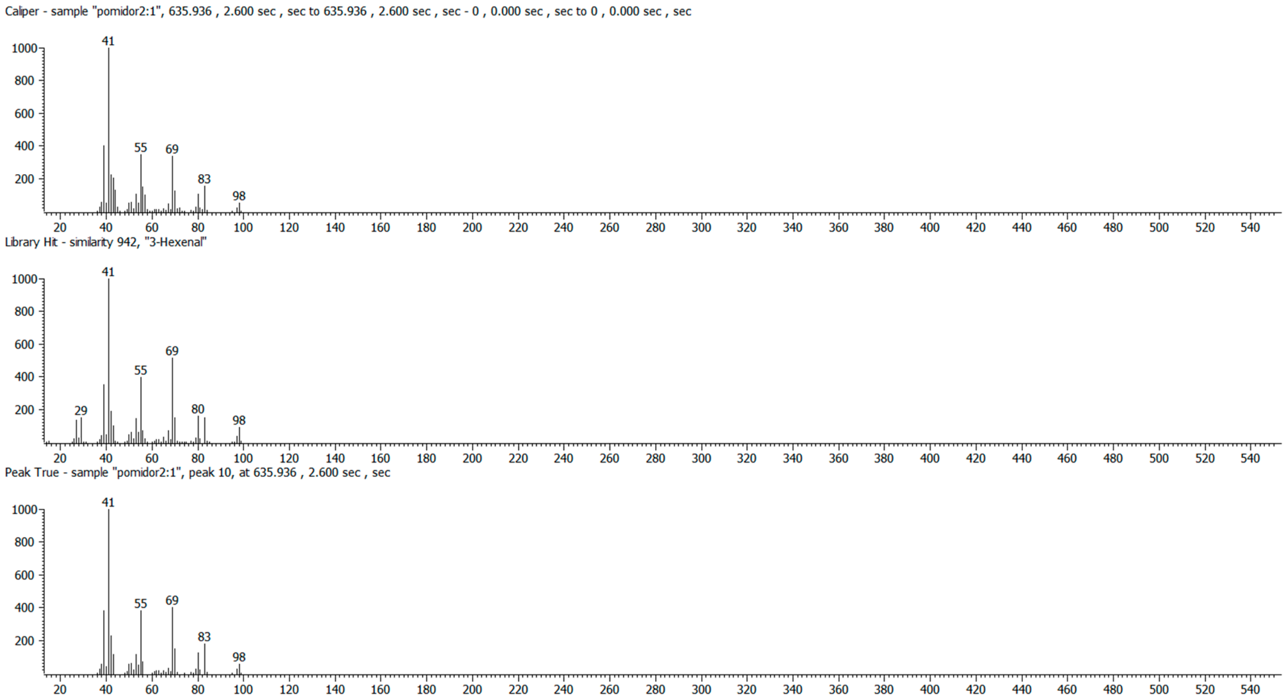
Caliper - sample "pomidor2:1", 635.936 , 2.600 sec , sec to 635.936 , 2.600 sec , sec - 0 , 0.000 sec , sec to 0 , 0.000 sec , sec
41
55
69
83
98
Library Hit - similarity 942, "3-Hexenal"
29
41
55
69
80
98
Peak True - sample "pomidor2:1", peak 10, at 635.936 , 2.600 sec , sec
41
55
69
83
98

(E)

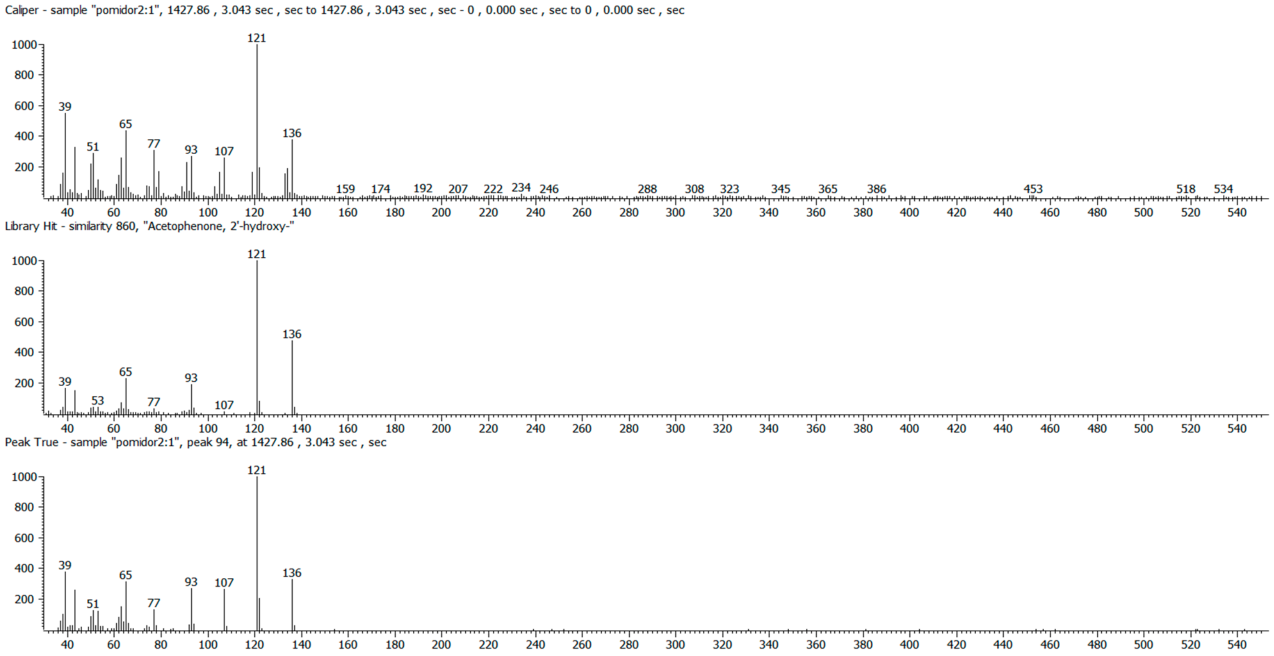
Caliper - sample "pomidor2:1", 1427.86 , 3.043 sec , sec to 1427.86 , 3.043 sec , sec - 0 , 0.000 sec , sec to 0 , 0.000 sec , sec
39
51
65
77
93
107
121
136
159 174 192 207 222 234 246 288 308 323 345 365 386 453 518 534
Library Hit - similarity 860, "Acetophenone, 2'-hydroxy-"
39
53
65
77
93
107
121
136
Peak True - sample "pomidor2:1", peak 94, at 1427.86 , 3.043 sec , sec
39
51
65
77
93
107
121
136

(F)

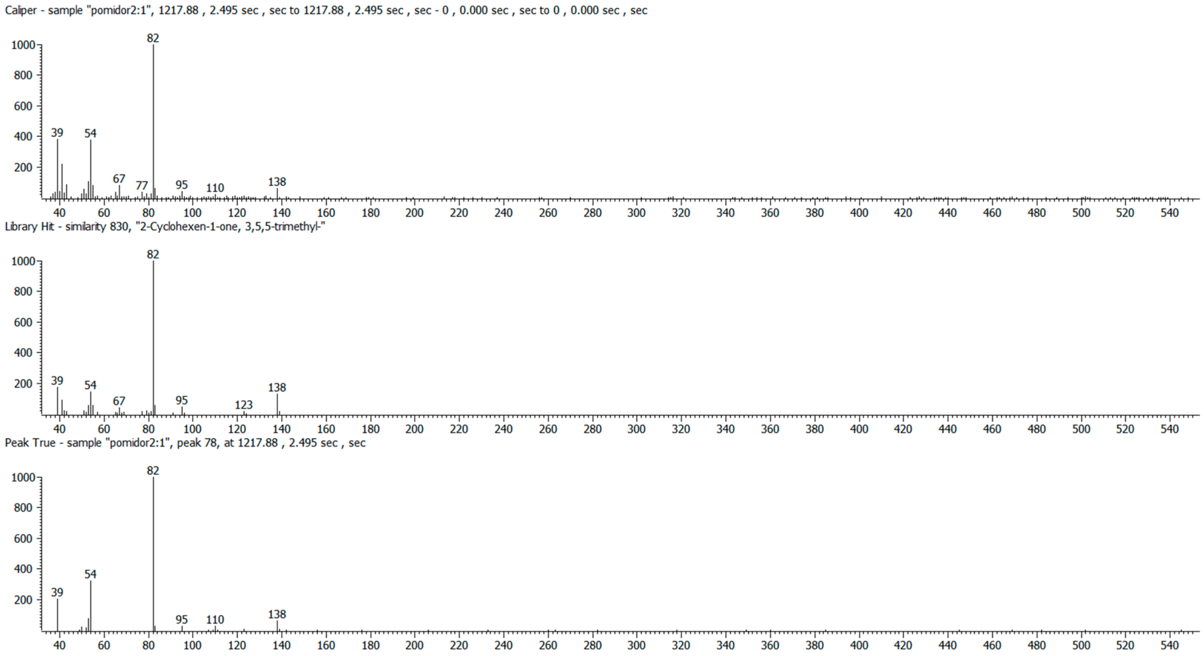
Caliper - sample "pomidor2:1", 1217.88 , 2.495 sec , sec to 1217.88 , 2.495 sec , sec - 0 , 0.000 sec , sec to 0 , 0.000 sec , sec
82
39
54
67
77
95
110
138
Library Hit - similarity 830, "2-Cyclohexen-1-one, 3,5,5-trimethyl-"
82
39
54
67
95
123
138
Peak True - sample "pomidor2:1", peak 78, at 1217.88 , 2.495 sec , sec
82
54
39
95
110
138

(G)

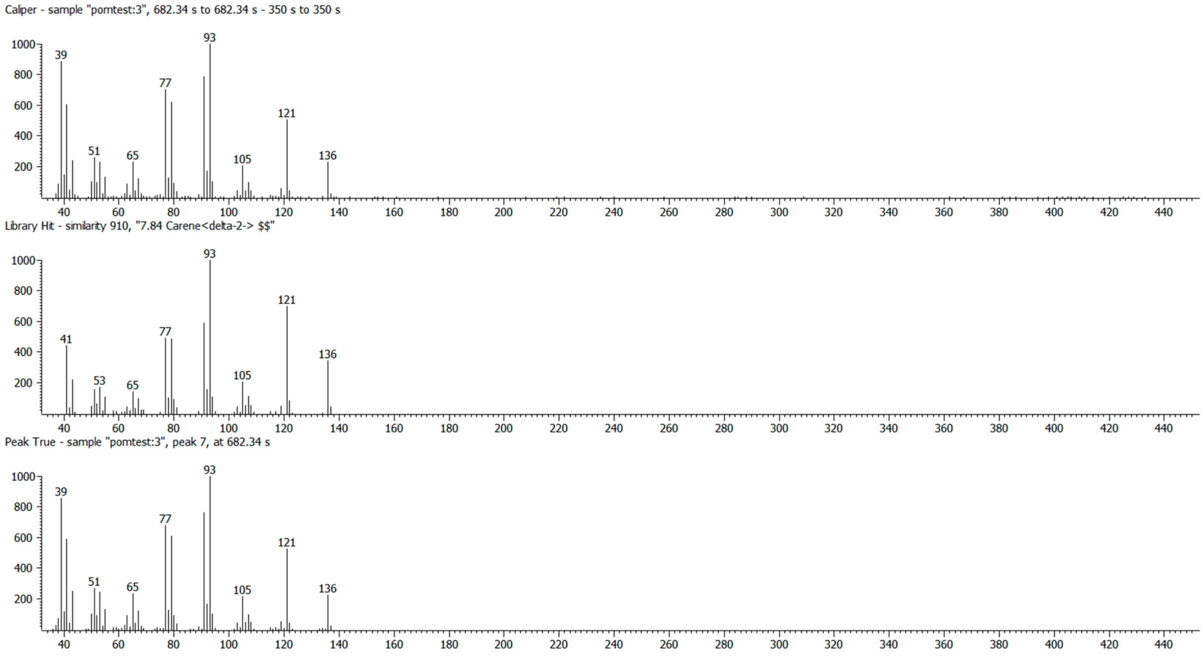
Caliper - sample "pomtest:3", 682.34 s to 682.34 s - 350 s to 350 s
93
39
77
121
51
65
105
136
Library Hit - similarity 910, "7.84 Carene<delta-2-> $$"
93
121
77
41
136
53
65
105
Peak True - sample "pomtest:3", peak 7, at 682.34 s
93
39
77
121
51
65
105
136

(H)

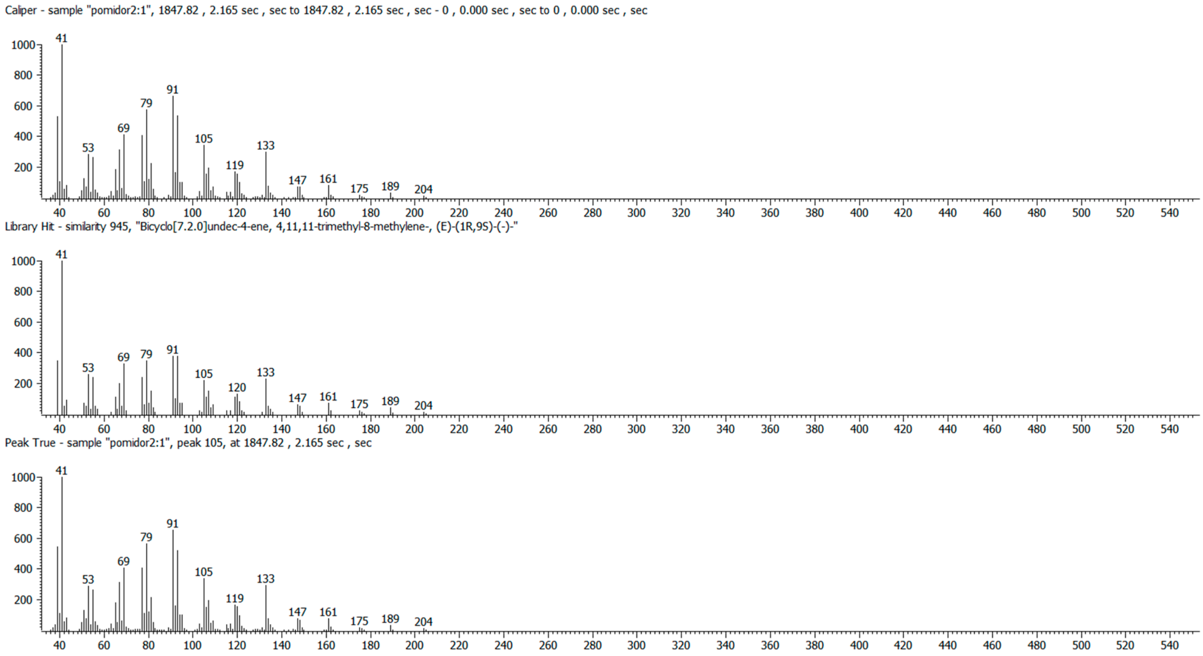
Caliper - sample "pomidor2:1", 1847.82 , 2.165 sec , sec to 1847.82 , 2.165 sec , sec - 0 , 0.000 sec , sec to 0 , 0.000 sec , sec
Library Hit - similarity 945, "Bicyclo[7.2.0]undec-4-ene, 4,11,11-trimethyl-8-methylene-, (E)-(1R,9S)-(-)-"
Peak True - sample "pomidor2:1", peak 105, at 1847.82 , 2.165 sec , sec

(I)

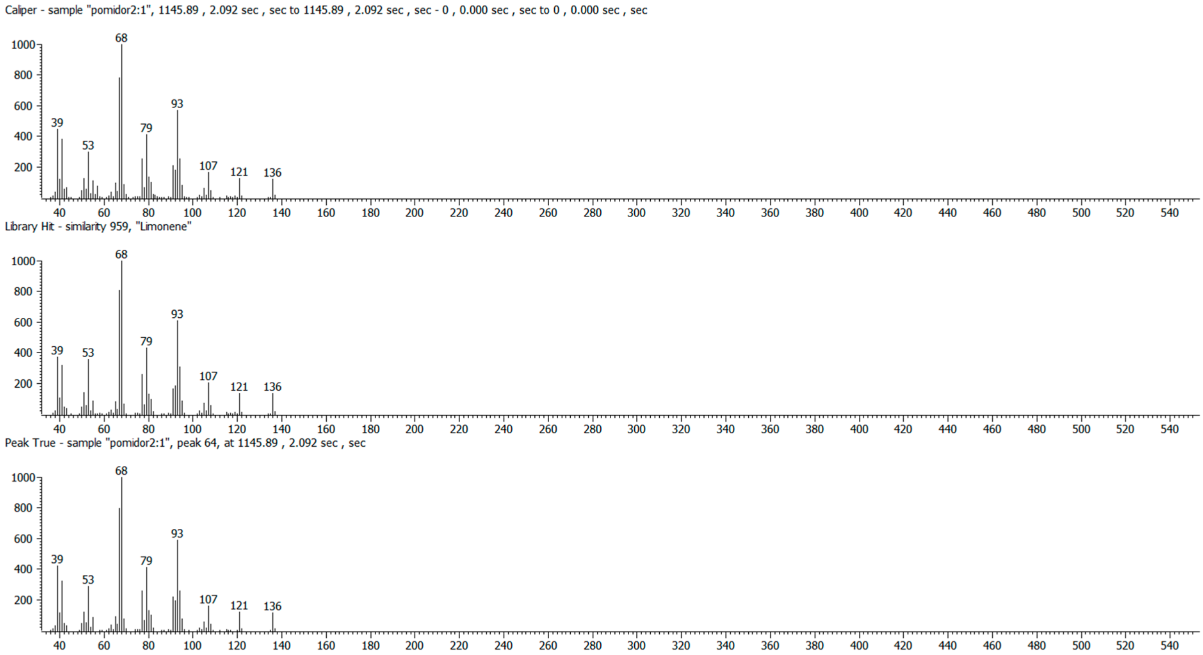
Caliper - sample "pomidor2:1", 1145.89 , 2.092 sec , sec to 1145.89 , 2.092 sec , sec - 0 , 0.000 sec , sec to 0 , 0.000 sec , sec
Library Hit - similarity 959, "Limonene"
Peak True - sample "pomidor2:1", peak 64, at 1145.89 , 2.092 sec , sec

(J)

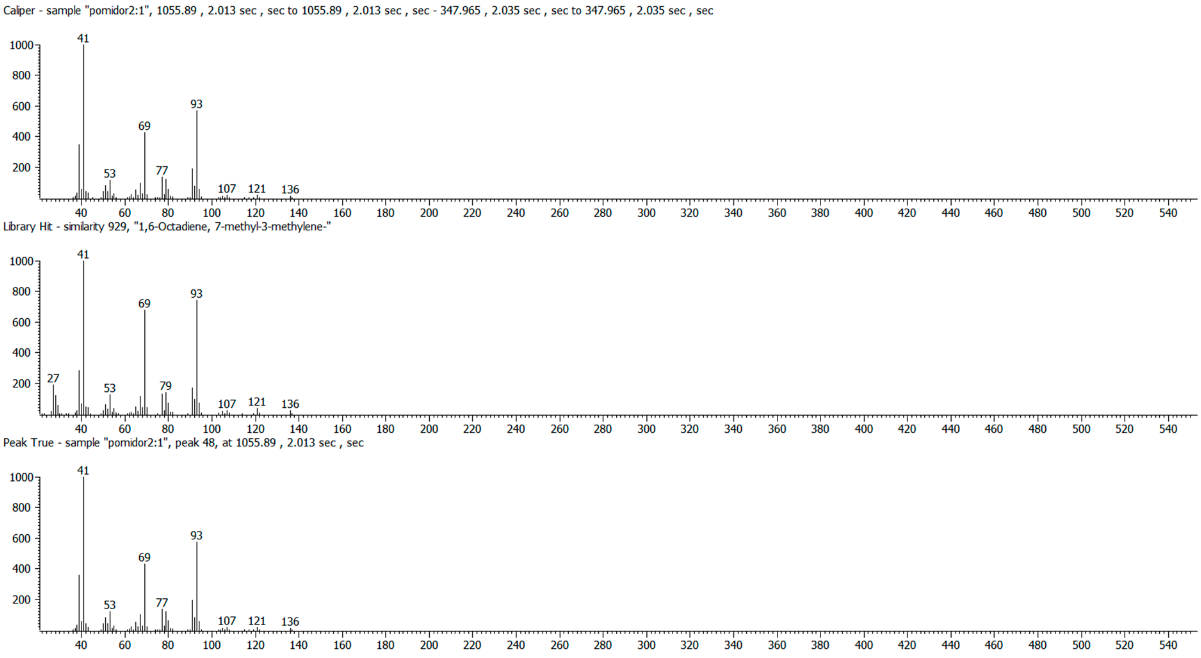

(K)

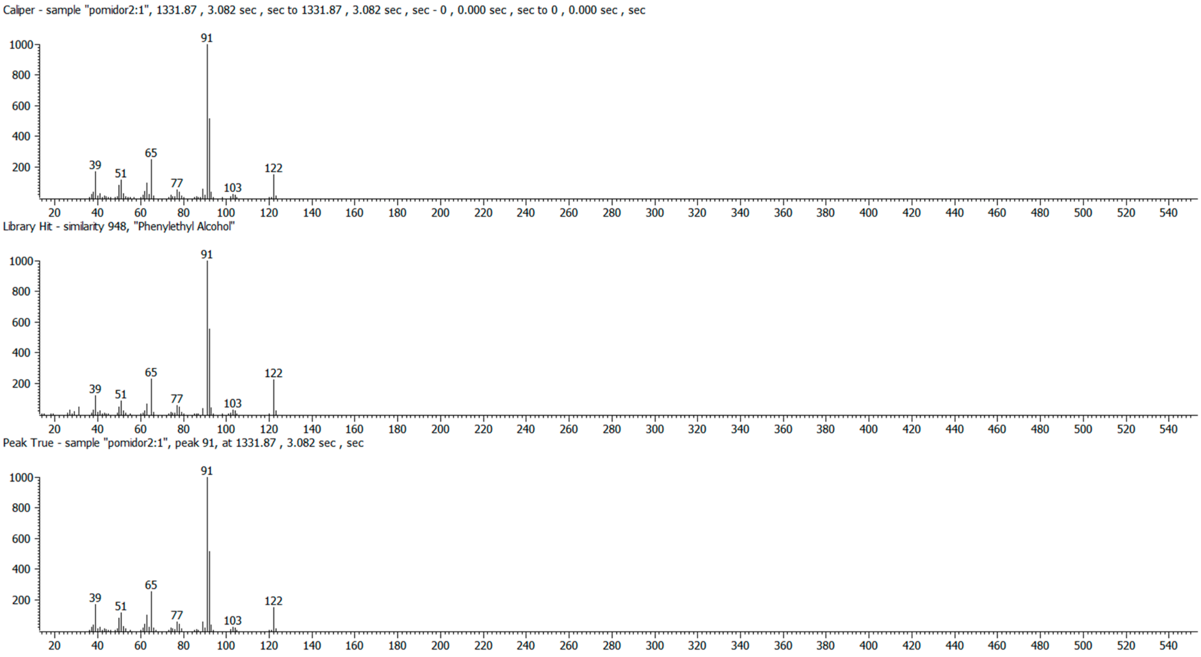

(L)

Figure S3. Representative mass spectra for exemplary VOCs; Hexanal **(A)**, α-Phellandrene **(B)**, 2-Methylphenol **(C)**, 2-Hexenal **(D)**, 3-Hexenal **(E)**, 2-Hydroxyacetophenone **(F)**, 3,3,5-Trimethy-2-cyclohexen-1-one **(G)**, 2-Carene **(H)**, Isocaryophyllene **(I)**, Limonene **(J)**, Myrcene **(K)**, Phenylethanol **(L)**.

Table S1. Selected VOCs annotated and putatively annotated in the tomato leaves. The identified compounds contain RI/MS/S identification. Abbreviations: VOC, volatile organic compound, RI_{exp}, relative retention indices calculated against n-alkanes (C_8-C_{20}) on BPX-5 column; RI_{lit}, relative retention indices on non-polar column (BPX-5 or similar stationary phase) reported in the literature (webbook.nist.gov); MS, NIST and Wiley libraries spectra; S, internal standard.

VOC	RI_{exp}	RI_{lit}	Deviation from reference (%)	Identification
**Alcohols**				
1-Penten-3-ol	688	688	0.0	RI/MS
4-Methyl-3-hexanol	721	721	0.0	RI/MS
2-Penten-1-ol	773	773	0.0	RI/MS
Prenol	778	778	0.0	RI/MS
Cyclopentanol	781	781	0.0	RI/MS/S
1-Pentanol	783	783	0.0	RI/MS/S
2-Hexanol	803	803	0.0	RI/MS
2-Hexyn-1-ol	851	847	-0.5	RI/MS
2-Hexen-1-ol	868	868	0.0	RI/MS
1-Hexanol	885	885	0.0	RI/MS/S
1-Heptanol	970	970	0.0	RI/MS/S
1,5-Octadien-3-ol	981	977	-0.4	RI/MS
7-Octen-4-ol	987	983	-0.4	RI/MS
1-Octen-3-ol	987	987	0.0	RI/MS/S
Phenol	992	992	0.0	RI/MS
6-Methyl-5-hepten-2-ol	993	993	0.0	RI/MS
Isooctanol	1002	1002	0.0	RI/MS/S
2-Ethyl-1-hexanol	1030	1030	0.0	RI/MS
2-methylphenol	1045	1042	-0.3	RI/MS
1-Octanol	1072	1072	0.0	RI/MS
2-methoxyphenol	1104	1102	-0.2	RI/MS
2-Nonen-1-ol	1105	1105	0.0	RI/MS/S
Phenylethanol	1132	1130	-0.2	RI/MS/S
**Aldehydes**				
Pentanal	698	698	0.0	RI/MS
2-Pentenal	710	710	0.0	RI/MS
2-Methyl-4-pentenal	798	798	0.0	RI/MS
Hexanal	810	810	0.0	RI/MS/S
3-Hexenal	818	818	0.0	RI/MS
2-Hexenal	832	832	0.0	RI/MS/S
(Z)-4-Heptenal	904	904	0.0	RI/MS
(2E,4E)-2,4-Hexadienal	912	911	-0.1	RI/MS
Heptanal	915	915	0.0	RI/MS
(E)-2-Heptenal	957	957	0.0	RI/MS
(E)-4-Oxohex-2-enal	963	958	-0.5	RI/MS
Benzaldehyde	964	964	0.0	RI/MS/S
Octanal	1003	1003	0.0	RI/MS/S
(Z)-6-Nonenal	1104	1101	-0.3	RI/MS
(2E,4E)-2,4-Heptadienal	1011	1009	-0.2	RI/MS
2-Hydroxybenzaldehyde	1043	1041	-0.2	RI/MS
2-Octenal	1060	1060	0.0	RI/MS
2,6-Dimethyl-5-heptenal	1063	1056	-0.7	RI/MS/S

4-Methylbenzaldehyde	1080	1079	-0.1	RI/MS
Nonanal	1163	1163	0.0	RI/MS
(E,Z)-2,6-Nonadienal	1166	1165	-0.1	RI/MS
4-Ethylbenzaldehyde	1177	1175	-0.2	RI/MS
3-Thujen-10-al	1197	1190	-0.6	RI/MS
Decanal	1203	1203	0.0	RI/MS
**Ketones**				
1-Penten-3-one	680	680	0.0	RI/MS
3-Pentanone	705	705	0.0	RI/MS
5-Methyl-2-hexanone	864	862	-0.2	RI/MS
5-Ethyl-2(5H)-furanone	963	963	0.0	RI/MS
6-Methyl-2-heptanone	966	962	-0.4	RI/MS
1-Octen-3-one	983	986	0.3	RI/MS
6-Methyl-5-hepten-2-one	994	994	0.0	RI/MS/S
2,2,6-Trimethyl-cyclohexanone	1051	1047	-0.4	RI/MS
Acetophenone	1073	1073	0.0	RI/MS
Ketoisophorone	1147	1142	-0.4	RI/MS
2-Hydroxyacetophenone	1149	1144	-0.4	RI/MS
3,6,6-Trimethyl-2-cyclohexen-1-one	1168	1166	-0.2	RI/MS
**Esters**				
Methyl acetate	536	536	0.0	RI/MS
Ethyl acetate	613	613	0.0	RI/MS
Butyl acetate	815	815	0.0	RI/MS/S
Hexyl acetate	1019	1019	0.0	RI/MS/S
Butyl hexanoate	1185	1186	0.1	RI/MS/S
Methyl salicylate	1235	1234	-0.1	RI/MS
Ethyl salicylate	1280	1279	-0.1	RI/MS
Isoamyl salicylate	1547	1542	-0.3	RI/MS
**Terpenoids**				
α-Pinene	937	937	0.0	RI/MS/S
(-)-Sabinene	976	976	0.0	RI/MS
β-Pinene	983	983	0.0	RI/MS/S
β-Myrcene	992	992	0.0	RI/MS/S
α-Phellandrene	1005	1005	0.0	RI/MS
2-Carene	1012	1010	-0.2	RI/MS
α-Terpinene	1017	1017	0.0	RI/MS
Limonene	1020	1020	0.0	RI/MS/S
o-Cymene	1022	1022	0.0	RI/MS
p-Cymene	1027	1026	-0.1	RI/MS/S
m-Cymene	1032	1030	-0.2	RI/MS
β-Phellandrene	1032	1032	0.0	RI/MS/S
β-Ocimene	1034	1031	-0.3	RI/MS
α-Ocimene	1044	1041	-0.3	RI/MS
γ-Terpinene	1062	1062	0.0	RI/MS
Cryptone	1083	1083	0.0	RI/MS
Terpinolene	1088	1088	0.0	RI/MS
β-cyclocitral	1228	1223	-0.4	RI/MS/S
δ-Elemene	1337	1337	0.0	RI/MS
α-Copaene	1376	1376	0.0	RI/MS
Isocaryophyllene	1431	1424	-0.5	RI/MS
α-Caryophyllene	1452	1452	0.0	RI/MS/S
β-Ionone	1489	1489	0.0	RI/MS/S

**Other VOCs**				
Acetic acid (Carboxylic acid)	599	599	0.0	RI/MS
Hexane (Alkane)	600	600	0.0	RI/MS
cis-1,2-dimethyl-cyclopentane (Alkane)	685	685	0.0	RI/MS
Naphthalene (Bicyclic aromatic hydrocarbon)	1178	1179	0.1	RI/MS
Azulene (Bicyclic aromatic hydrocarbon)	1314	1311	-0.2	RI/MS
2-Methyltetrahydrofuran (Heterocyclic compound)	678	678	0.0	RI/MS
2-Ethylfuran (Heterocyclic compound)	702	702	0.0	RI/MS
2-Pentylfuran (Heterocyclic compound)	993	993	0.0	RI/MS
2-Ethylthiophene (Heterocyclic compound)	871	871	0.0	RI/MS
Benzofuran (Heterocyclic compound)	1015	1015	0.0	RI/MS

Table S2. Volatile metabolites annotated and putatively annotated in the volatile profiles of *B. cinerea* present on the tomato leaves **(A)** and *T. virens* TRS 106 present in the tomato rhizosphere **(B)**.

Abbreviations: VOC, volatile organic compound, RI_{exp}, relative retention indices calculated against n-alkanes (C_8-C_{20}) on BPX-5 column; RI_{lit}, relative retention indices on non-polar column (BPX-5 or similar stationary phase) reported in the literature (webbook.nist.gov); MS, NIST and Wiley libraries spectra; S, internal standard.

(A)

VOC	RI_{exp}	RI_{lit}	Deviation from reference (%)	Identification
Ethanol	450	450	0.0	RI/MS/S
Hexane	600	600	0.0	RI/MS/S
Ethyl acetate	613	613	0.0	RI/MS/S
1-Octen-3-ol	987	987	0.0	RI/MS/S

(B)

VOC	RI_{exp}	RI_{lit}	Deviation from reference (%)	Identification
3-Heptanone	896	896	0.0	RI/MS
Octan-3-one	996	998	0.2	RI/MS
3-Octanol	998	1001	0.3	RI/MS
2,6-Dimethylnonane	1019	1022	0.3	RI/MS
2-Ethyl-1-hexanol	1035	1038	0.3	RI/MS
Unknown	1045	-	-	-
Dihydromyrcenol	1075	1075	0.0	RI/MS
Viridiflorene	1510	1511	0.1	RI/MS
Ledol	1590	1593	0.2	RI/MS
Farnesol	1688	1690	0.1	RI/MS
